# Yangke powder alleviates OVA-induced allergic asthma by inhibiting the PI3K/AKT/NF-κB signaling pathway

**DOI:** 10.1186/s13020-025-01125-x

**Published:** 2025-05-26

**Authors:** Xueyan Li, Lu Ding, Zirui Li, Zhenghua Cao, Min Li, Kai Yin, Siyu Song, Liyuan Cao, Qinjing Xia, Zihan Wang, Daqing Zhao, Xiaolin Tong, Xiangyan Li, Zeyu Wang

**Affiliations:** 1 Affiliated Hospital of Changchun University of Chinese Medicine, Changchun, 130021 Jilin China; 2 Research Center of Traditional Chinese Medicine, The Affiliated Hospital of Changchun University of Chinese Medicine, Changchun, 130021 Jilin China; 3 Northeast Asia Research Institute of Traditional Chinese Medicine, Changchun University of Chinese Medicine, Changchun, 130021 China; 4 Institute of Metabolic Diseases, Guang’anmen Hospital, China Academy of Chinese Medical Sciences, Beijing, 100053 China

**Keywords:** Yangke powder, Asthma, Inflammation, UPLC-Q-exactive orbitrap-MS, Network Pharmacology, Molecular Docking

## Abstract

**Background:**

Asthma is a chronic inflammatory airway disease that remains inadequately controlled by existing conventional treatments. A traditional Chinese medicine (TCM) formula of Yangke powder (yǎng ké sǎn-YKS) has demonstrated potential in alleviating asthma symptoms and reducing its acute exacerbation. Despite clinical evidence supporting its benefit, there is still insufficient understanding of the active compounds in YKS and their underlying mechanisms, which limits its broader clinical application.

**Objective:**

This study aims to identify the key active ingredients in YKS and explore their mechanisms, particularly through the PI3K/AKT/NF-κB pathways, to provide a scientific basis for its application in asthma treatment.

**Methods:**

We employed UPLC-Q-Exactive Orbitrap-MS to analyze YKS constituents, identified key ingredients, and explored asthma treatment mechanisms through bioinformatics, network pharmacology, Mendelian randomization, and molecular docking. The asthma model was evaluated using ovalbumin (OVA) and pulmonary function tests, while pathological examination was conducted using hematoxylin and eosin (HE), periodic acid-Schiff (PAS), and Masson trichrome stains. Concentrations of IgE, IL-4, and IL-5 were measured by ELISA, and protein and mRNA expressions were confirmed via qPCR, immunohistochemistry, and Western blot analysis.

**Results:**

A total of 174 compounds were identified in YKS by UPLC-MS, with 49 detected in the bloodstream, indicating their role as active ingredients. Bioinformatics analysis revealed 353 asthma-related targets and 972 potential targets for YKS. Key targets such as AKT1, TNF, and IL1B were validated by molecular docking. Our studies indicated that YKS modulates asthma primarily through the PI3K/Akt and NF-κB pathways, improving airway resistance, reducing inflammation, mucus production, and airway remodeling, and decreasing Th2 cytokines and IgE levels.

**Conclusion:**

This investigation identifies Kaempferol, Norephedrine, Cynaroside, Genistein, and Rutin as critical active ingredients in YKS, impacting key biomarkers such as AKT1, TNF, and IL1B. These substances effectively modulate the PI3K/AKT/NF-κB pathway, enhancing the management of allergic asthma.

**Supplementary Information:**

The online version contains supplementary material available at 10.1186/s13020-025-01125-x.

## Introduction

Asthma is a heterogeneous disorder defined by chronic, reversible inflammation of the airways. It presents with respiratory symptoms that vary in severity and frequency, along with variable airflow obstruction [1]. Asthma poses a significant global health challenge, affecting all age groups [2]. Its pathogenesis is driven by a complex interplay of genetic and environmental factors [3], resulting in hundreds of millions of cases worldwide [4]. As a leading contributor to the global disease burden [5] asthma is recognized as a primary cause of morbidity and mortality [6]. The primary goals of current asthma therapies focus on minimizing symptom severity and reducing the incidence of severe exacerbations, emphasizing symptom management and halting disease progression [7]. However, a universally accepted definition of asthma remains elusive [8].

Contemporary asthma pharmacotherapies are noted for their wide array of side effects, which can deter patient compliance and lead to recurrent episodes [9]. Moreover, these treatments may induce structural changes in the airways, potentially accelerating the decline in lung function [10]. Traditional Chinese Medicine (TCM) has shown advantages in preventing or treating respiratory conditions [11] and has been adopted by 70% of U.S. asthma patients who incorporate herbal remedies into their treatment plans [12]. Evidenced by the growing clinical adoption of these herbal strategies [13], systematic reviews have documented and affirmed their ability to reduce airway remodeling, protect against asthma-related pulmonary deterioration, and improve treatment outcomes [14]. These herbal treatments improve lung capacity [15], complement existing asthma therapies [16], and alleviate asthma-related symptoms (e.g., airway inflammation and excess mucus production) [17], offering advantages such as reduced side effects, minimal toxicity, and no systemic immunosuppressive effects [18].

**Fig. 1:**
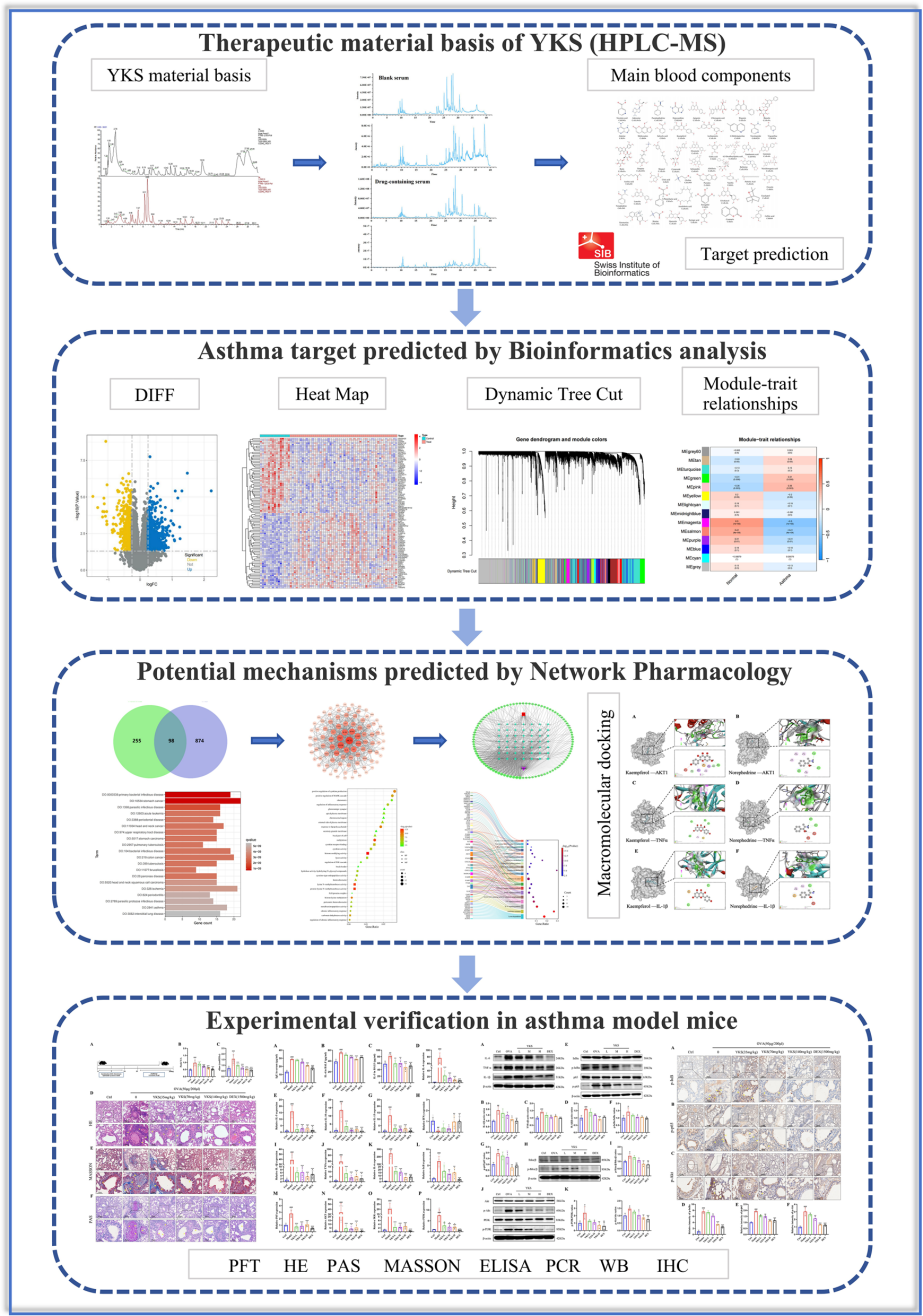
Research process

YangKe powder (yǎng ké sǎn-YKS), with a blend of thirteen specific herbs (Table 1), is formulated under the direction of Professor Xiaolin Tong from the Chinese Academy of Sciences, integrating traditional knowledge from the *She Gan Ma Huang Tang* formula with contemporary clinical practices. Clinical studies have shown that YKS could also decrease the frequency of severe exacerbations and enhance the overall patient well-being, in addition to ameliorating asthma symptoms. Despite these advances, the active compounds and their precise mechanisms within YKS have yet to be fully identified. To address this, the current study intended to analyze both the lyophilized YKS extract and post-gavage medicated plasma using cutting-edge UPLC-Q-Exactive Orbitrap-MS technology, integrating several research methods including bioinformatics, network pharmacology, Mendelian randomization (MR), and in vivo studies. Through such thorough investigation, this study is expected to elucidate the mechanisms by which YKS alleviates allergic airway inflammation, thus providing a strong scientific basis for further exploration of the therapeutic potential of YKS in asthma management (Fig. 1).

**Table 1:** The detailed information of YKS’s compositions

Chinese name	Drug name	Abbr	Family	Part used	Voucher specimen
She–gan	Blackberrylily rhizome	SG	Iridaceae	Rhizome	20,230,801–01
Zhi-ma-huang	Ephedra herb	ZMH	Ephedraceae	Herbaceous stems	20,230,801–02
Gan-jiang	Zingiberis rhizoma	GJ	zingiberaceae	Rhizome	20,230,801–03
Qing-ban-xia	Pinelliae Rhizoma	QBX	Araceae	Rhizome	20,230,801–04
Zhi-zi-wan	Tatarian Aster Root	ZZW	Asteraceae	Rhizome	20,230,801–05
Zhi-kuan-dong-hua	Farfarae Flos	ZKDH	Asteraceae	Flower bud	20,230,801–06
Pi-pa-ye	Eriobotryae folium	PPY	Rosaceae	Leaf	20,230,801–07
Wu-wei-zi	Schisandra chinensis	WWZ	Magnoliaceae	Fruit	20,230,801–08
Qian-hu	Peucedani Radix	QH	Umbelliferae	Root	20,230,801–09
Bai-bu	Stemonae Radix	BB	Stemonaceae	Rhizome	20,230,801–10
Zi-su-zi	Perillae fructus	ZSZ	Lamiaceae	Fruit	20,230,801–11
Ting-li-zi	Descurainiea semen	TLZ	Lepidium apetalum Willd	Seed	20,230,801–12
Di-long	Pheretima	DL	Pheretima aspergillum	All	20,230,801–13

## Methods and materials

### Animal and ethical approval

Animal experiments, approved by the Medical Ethics Committee of Changchun University of Chinese Medicine (Approval No: 2024836), were carried out in accordance with the Guidelines for the Care and Use of Laboratory Animals. Female BALB/c mice, aged 8–10 weeks and weighing 20–25 g, were sourced from Changchun Yisi Biotech, Jilin, China [license No: SCXK (Ji) 2020–0002], and male specific pathogen-free (SPF) Sprague–Dawley (SD) rats, aged 6–8 weeks and weighing 180–220 g, were obtained from Liaoning Changsheng Biotechnology [license No.: SCXK (Liaoning) 2020–0001]. Prior to the commencement of the experiments, animals were allowed to acclimate for one week in a pathogen-free environment maintained at 22 ± 1 °C and 50 ± 5% humidity, with a 12-h light/dark cycle. Standard feed was provided throughout the acclimation and experimental periods.

### Chemicals and reagents

Methanol of chromatography quality was sourced from Thermo Fisher Scientific (China) Co., Ltd. Formic acid (chromatography grade) was provided by Shanghai Aladdin Biochemical Technology Co., Ltd. Ovalbumin (OVA, A5503-10G) and Aluminum Hydroxide (239,186-25G) was obtained from Sigma-Aldrich (St. Louis, MO, USA). The PAS staining kit (catalog number G1281) and the MASSON staining kit were acquired from Beijing Solarbio Science & Technology Co., Ltd. The Mouse IgE ELISA kit (PMLB00 C) was purchased from Shanghai Enzyme-linked Biotechnology Co., Ltd., which also supplied the Mouse IL-4 ELISA kit (RK00036) and Mouse IL-5 ELISA kit (RK00037). Antibodies including phospho-NF-κB p65 (BS66162), IKKα/β (BZ16443), and phospho-IKKα/β (BZ16321) were procured from Bioworld Technology, China. Proteintech Group, Wuhan, China, provided the IL-6 antibody (26,404–1-AP), IL-1β antibody (26,048–1-AP), TNFα antibody (17,590–1-AP), β-Actin antibody (66,009–1-Ig), NF-κB p65 antibody (80,979–1-RR), IκB antibody (66,418–1-Ig), and horseradish peroxidase (HRP)-conjugated Goat Anti-mouse (SA00001-1) and Goat Anti-rabbit (SA00001-2) antibodies. ABMART, China, supplied the phospho-IκB antibody (TP56280), phospho-AKT antibody (T40067), AKT antibody (T55561), phospho-PI3 K antibody (T40116), and PI3 K antibody (T40115). The thirteen traditional Chinese medicinal components of YKS, adhering to Chinese Pharmacopoeia standards, were provided by the pharmacy of the Affiliated Hospital of Changchun University of Chinese Medicine.

### Identification of the material basis of YKS in vitro and in vivo

#### Preparation of YKS

Before preparation, the thirteen selected traditional Chinese medicinal herbs composing YKS were immersed in distilled water (water: herbs = 10:1, v/v) and allowed to soak for 60 min. After the initial boiling phase for 30 min, the mixture was promptly strained while still hot. The remaining insoluble residue was then mixed with water (water: herbs = 5:1, v/v) and subjected to a second boiling (100 °C, 30 min). After the second filtration, the leftover residue was mixed with 2 volumes of water (water: herbs = 2:1 v/v) for a final decoction. The liquid extracts from all three boiling stages were mixed and concentrated into a dense paste, which was solidified at −20 °C for 12 h. The solidified paste is then freeze-dried under vacuum conditions at −80 °C, resulting in the production of lyophilized YKS powder. Exactly 0.1 g of freeze-dried YKS powder was mixed with 1 mL of 80% (v/v) methanol in a tube containing grinding beads. The mixture was then ground for 5 min, subjected to vortex mixing for ten minutes, and subsequently centrifuged at 13,000 rpm for 10 min. The resulting supernatant was collected and passed through a 0.22 µm microporous membrane filter to yield the final YKS extract.

### Collection of YKS components in blood

A total of ten male SPF-grade SD rats were randomly assigned to either a control group or a treatment group using a random number table method. The treatment group received oral administration of YKS at a dosage of 9.04 g/kgBW, equivalent to four times the standard clinical oral dose for adult humans, appropriately scaled for the rats'weight. In contrast, the control group was provided with distilled water following the same administration protocol. After three consecutive days of treatment, the rats were fasted for 12 h before the final dose. Blood samples were collected from the jugular vein of anesthetized rats at 0.5, 1, 2, and 4 h after the last administration. The collected blood was transferred into heparinized centrifuge tubes and then centrifuged at 4,500 rpm for 10 min at 4 °C. The resulting supernatant was extracted, and the serum from the treatment group was pooled to create two separate serum samples. These serum aliquots were subsequently divided and stored at −80 °C for future analyses.

### Mass spectrometry and chromatography conditions

The ionization process employed Electrospray Ionization (ESI) as the source, with the instrument configured to alternate scanning modes between positive and negative ions. Detection utilized both full mass scans and data-dependent MS^2^ (dd-MS^2^) modes. The mass analyzer achieved a resolution of 70,000 for full mass scans and 17,500 for dd-MS^2^. The mass-to-charge (m/z) range was set from 100.0 to 1500.0. The electrospray was maintained at a voltage of 3.2 kilovolts for both ionization modes, and the capillary temperature was held at 300 °C. High-purity argon gas (purity ≥ 99.999%) was used as the collision gas, with collision energies set at Normalized Collision Energy (NCE) levels of 30, 40, and 60. Nitrogen gas (purity ≥ 99.999%) functioned as both the sheath gas, at a flow rate of 40 arbitrary units (Arb), and the auxiliary gas, at 15 Arb with a temperature of 350 °C. Data acquisition spanned a duration of 30 min. Chromatographic separation was accomplished using an AQ-C18 column, 150 mm by 2.1 mm with a particle size of 1.8 µm, from Welch. The flow rate was maintained at 0.30 mL. The mobile phase consisted of an aqueous solution with 0.1% formic acid and methanol as the organic phase. The column oven temperature was set at 35 °C, while the autosampler was maintained at 10.0 °C. Each sample injection volume was precisely 5.00 µL.

### Data processing

Initially, a comprehensive database for YKS was established by integrating data from the Traditional Chinese Medicine Systems Pharmacology Database and Analysis Platform (TCMSP) and relevant scholarly articles. This database was organized to include information on the in vitro constituents of YKS as well as its components found in blood, utilizing CD3.3 software (Thermo Fisher). Initial identification of compounds was performed by comparing the collected data with the MzCloud database and an internal proprietary database. Subsequently, specific compounds were selected based on their retention times and fragment ion patterns, with a strict selection error threshold of 10 parts per million (ppm).

### Network pharmacology research

#### Confirmation of asthma disease targets

Initially, the GSE137268 dataset was retrieved from the Gene Expression Omnibus (GEO) database. This dataset contains gene expression data from 15 healthy individuals and 54 patients diagnosed with asthma. This dataset underwent normalization to correct for technical variations and ensure consistency across all samples. Following normalization, genes exhibiting significant differential expression were identified using stringent criteria, specifically an absolute |log fold change (logFC)| exceeding 0.25 and a p-value below 0.05. These asthma-associated differentially expressed genes (DEGs) were then analyzed through Gene Set Enrichment Analysis (GSEA) to uncover relevant biological pathways and processes. Concurrently, the same GSE137268 dataset was subjected to Weighted Gene Co-expression Network Analysis (WGCNA) to construct a scale-free co-expression network. Through hierarchical clustering within this network, specific gene modules related to asthma were identified. The pivotal genes from these asthma-related modules were subsequently intersected with the previously identified DEGs to pinpoint potential target genes implicated in the pathogenesis of asthma.

#### Collection of target sites for the active blood components of YKS

The PubChem database (https://pubchem.ncbi.nlm.nih.gov/) was searched to retrieve 49 active blood components of YKS to obtain their SDF file formats and Canonical SMILES representations. To identify potential biological targets, these molecular entities were subsequently entered into the Swiss Target Prediction platform (http://www.swisstargetprediction.ch/, STP) [19]. Finally, the functional targets associated with the blood components of YKS were determined by removing redundant entries.

#### Potential targets, PPI, enrichment analysis, and network construction for YKS treatment of asthma

The initial step was to establish the intersection between the active blood constituents of YKS and potential asthma-related target genes. The identified common genes were then submitted to the STRING database (https://string-db.org/) to construct a Protein–Protein Interaction (PPI) network using specified parameters. To facilitate the identification of key central targets, the generated data were loaded into Cytoscape software to produce a network highlighting the primary targets visually. Simultaneously, a detailed network diagram integrating the blood components of YKS, their respective targets, and asthma was developed using Cytoscape to pinpoint the principal effective components.

Enrichment analyses were further performed using R software on the intersecting targets through Disease Ontology (DO), Gene Ontology (GO), and Kyoto Encyclopedia of Genes and Genomes (KEGG) databases. Subsequently, diagrams for DO, GO, and KEGG enrichment were generated and visualized to understand potential signaling pathways involved in YKS treatment of asthma and to explore the underlying mechanisms.

#### Molecular docking and Mendelian randomization analysis

Molecular docking validation was performed on key targets (IL1B, TNF, and AKT1) identified through PPI screening. From the network analysis, the potent active compounds Kaempferol and Norephedrine were selected for this validation process. MR, a technique for causal inference [20], was employed to verify the causal associations between specific genes and diseases [21]. Consequently, this study explored genetic causal relationships of IL1B (prot-a-1495), TNF (prot-a-3029), and AKT1 (eqtl-a-ENSG00000142208) with asthma (finn-b-ASTHMA_NAS_EXMORE) using data sourced from the GWAS database [22].

In evaluating exposure factors, association analyses were conducted with a strict significance threshold (P < 1 × 10⁻^5^). Subsequently, SNPs showing linkage disequilibrium with an R2R^2R2 value lower than 0.001, and located within a 10,000 Kb region were methodically excluded. To ensure robust instrumental variables, F-statistics were calculated for the remaining SNPs, and only those with F > 10 were retained to avoid weak instrument bias. The causal relationship with asthma was primarily analyzed using the inverse-variance weighted (IVW) method. Additionally, MR-Egger, weighted median, simple mode, and weighted mode were employed to assess heterogeneity, pleiotropy, and to perform sensitivity analyses.

### Animal experimentation

#### Mouse modeling and grouping

To initiate sensitization, on days 0, 7, and 14, mice received intraperitoneal injections of a solution with 50 μg of OVA dissolved in saline and enhanced with 1 mg of aluminum hydroxide powder. From day 21 to day 35, these mice were subjected to nebulization sessions (30 min per day) using a 2% OVA aerosol to challenge their immune systems. Mice in the control group received equivalent treatments with saline instead of OVA (Fig. 6A).

The experimental mice were divided into six distinct groups, each comprising five mice, with corresponding naming and treatment methods as follows: (1) Control (Con) group: received sterilized saline; (2) OVA group: treated with OVA in saline; (3) OVA + YKS (Low dose) group: asthmatic mice were administered YKS orally at a dose of 0.035 g/kg, 30 min prior to nebulization, daily from day 21 to day 35; (4) OVA + YKS (Medium dose) group: asthmatic mice received YKS at a dose of 0.07 g/kg, following the same administration schedule; (5) OVA + YKS (High dose) group: asthmatic mice were given YKS at a dose of 0.14 g/kg under identical conditions; (6) OVA + Dexamethasone (DEX) group: asthmatic mice were orally administered 1500 mg/kg of dexamethasone (Zhejiang Xianju Pharmaceutical, China) 30 min before each OVA nebulization session. On day 35, exactly 24 h after the final OVA nebulization, after humane euthanasia, we collected tissues from all mice, which concluded the experiment.

#### Non-invasive pulmonary function testing in mice

To assess respiratory function in mice, this study employed a non-invasive airway monitoring system (NAM, DSI Buxco) utilizing the plethysmographic method. Briefly, mice were placed within a specially designed plethysmographic chamber to measure specific airway resistance (sRaw). This measurement is derived by assessing the phase shift between airflow through the nasal passages and the airflow within the thoracic region. When using the whole-body plethysmography system (WBP, DSI Buxco), mice were housed in a chamber outfitted with highly sensitive pressure transducers, enabling the detection even the slightest pressure changes within the chamber. The minute airflow variations detected were then analyzed by the Buxco FinePointe software, which employs advanced algorithms and analytical methods to continuously monitor the extent of bronchoconstriction (PenH). The recording chamber maintains a time constant of 0.2 s, and each mouse is subjected to a 2-min recording session to ensure the acquisition of precise measurements.

#### Histological examination

Lung tissue samples were preserved by immersing them in a 4% paraformaldehyde solution for 30 min, and then they were embedded in paraffin and sectioned into slices 5 µm thick. Standard histological staining techniques were then applied, including Hematoxylin and Eosin (H&E) staining, Masson's trichrome staining, and Periodic Acid-Schiff (PAS) staining. The H&E staining was utilized to observe the structural changes in the lung tissues, which are indicative of bronchial inflammation. An arbitrary scoring system was employed for evaluation, conducted in a blinded manner using semi-quantitative scoring methods. Inflammation severity was categorized as follows: grade 0 indicated no inflammation; grade 1 for less than 25% inflammatory cells; grade 2 for 25–50% inflammatory cells; grade 3 for 50–75% inflammatory cells; and grades above 3 for inflammatory cells exceeding 75%. PAS staining was used to assess the proliferation of goblet cells, quantifying mucus production by measuring the percentage of PAS-positive cells within the airway epithelium. Through Masson's trichrome staining, we visualized collagen deposition within the bronchial airway and quantified it by calculating the proportion of the airway area exhibiting positive staining. For each tissue section, three fields were randomly selected and photographed at 200-fold magnification for detailed analysis.

#### ELISA

For ELISA, both serum samples and bronchoalveolar lavage fluid (BALF) were meticulously collected from the mice after the completion of the animal experiments. The BALF was prepared by conducting three separate lung lavage sessions via tracheal cannulation (to ensure efficient fluid collection), each using 0.5 mL of cold, sterile saline solution. After that, approximately 1.5 mL of the collected BALF was subjected to 10 min of centrifugation at 1,500 rpm at 4 ℃. This centrifugation was crucial for effectively separating the cellular pellets from the clear supernatant fluid. The quantification of the levels of IgE in the serum, as well as IL-4 and IL-5 in the BALF was realized using specific mouse IgE, IL-4, and IL-5 ELISA kits, respectively, in strict accordance with the protocols provided by the manufacturers.

### Reverse transcription and quantitative real-time polymerase chain reaction (qPCR)

Lung tissue samples were subjected to total RNA isolation using the Total RNA Extraction Kit (Beijing Tiangen Biotech Co., Ltd.). Concentration of the extracted RNA was precisely quantified using a UV spectrophotometer (NANO 2000, ThermoFisher, Waltham, MA, USA). Subsequently, exactly 1 µg of the isolated total RNA was reverse transcribed into cDNA using the iScript cDNA Synthesis Kit (Beijing Tiangen Biotech Co., Ltd.). For gene expression analysis, real-time qPCR was performed using the Bio-Rad CFX96 system (Bio-Rad, Synergy Mx). Then, the 2^-ΔΔCt^ method was employed to ensure accurate normalization of gene expression data, with β-ACTIN as the internal reference gene. All primers used in the qPCR were procured from Sangon Biotech (Shanghai) Co., Ltd. Details regarding the primer sequences and their respective target genes are available in Supplementary Table S1.

### Immunohistochemistry

Similar to the processing in histological examination, Sections measuring 5 µm thick were prepared, carefully mounted on polylysine-coated slides and placed in a 60 °C oven for 24 h to ensure optimal adhesion. After the removal of the paraffin using xylene, the next step was rehydration through a series of gradient alcohols. The slides underwent antigen retrieval by being exposed to a solution and high-powered microwave irradiation for 15 min. After cooling and washing with a buffer solution, the slides were sequentially incubated with a primary antibody and a biotin-conjugated secondary antibody. Then, after staining with DAB, these sections were counterstained with hematoxylin for contrast, followed by dehydration through an alcohol series, clearing with xylene, and mounting for microscopic examination. At 400 × magnification, three distinct microscopic fields were randomly selected for analysis. The expression levels of phosphorylated AKT (p-AKT), phosphorylated NF-κB p65 (p-NF-κB p65), and phosphorylated IκB (p-IκB) were quantitatively evaluated using ImageJ software, measuring the average optical density of areas showing positive staining.

### Western blot analysis

Lung tissue samples from various experimental groups were lysed using a sample buffer and then denatured to prepare the proteins for analysis. Protein concentrations were accurately quantified using the BCA Protein Assay Kit (Beyotime, China). To ensure uniformity, equal amounts of protein from each sample were subjected to SDS-PAGE for electrophoretic separation based on molecular weight. After transferring onto PVDF membranes. These membranes were blocked with a 5% bovine serum albumin (BSA) solution to prevent non-specific binding. Next, membranes were incubated overnight at 4 °C with primary antibodies targeting specific proteins. The targeted proteins included IL-6, IL-1β, TNFα (all diluted 1:1000), phosphorylated IκB α (p-IκB α, 1:500), I-κB α, NF-κB P65, phosphorylated NF-κB P65 (p-NF-κB P65), AKT, phosphorylated AKT (p-AKT), phosphorylated PI3K (P-PI3K), PI3 K (all diluted 1:1000), β-ACTIN (1:10,000), phosphorylated IKK (p-IKK), and IKK (both diluted 1:1000). After that, the membranes were exposed to HRP-conjugated secondary antibodies (either goat anti-rabbit IgG or goat anti-mouse IgG, diluted 1:5000) and incubated at room temperature on a shaker for 1 h. Protein band intensity was detected using enhanced chemiluminescence detection reagents (Beyotime, China) and normalized against β-ACTIN levels. Quantitative analysis of the blots was performed using ImageJ software, with all experiments conducted in triplicate to ensure reproducibility.

### Statistical analysis

All experimental measurements, generated from no fewer than three separate and independent experimental trials, were reported as mean values with their respective standard deviations (mean ± SD). An analysis of variance was performed to assess the statistical significance of inter-group differences. Bonferroni’s multiple comparison tests were further utilized to evaluate specific group differences. The analyses were conducted using GraphPad Prism 9.0, with a p-value threshold of less than 0.05 set to denote statistical significance.

## Results

### In vitro material basis and blood-entry component analysis of YKS

**Fig. 2:**
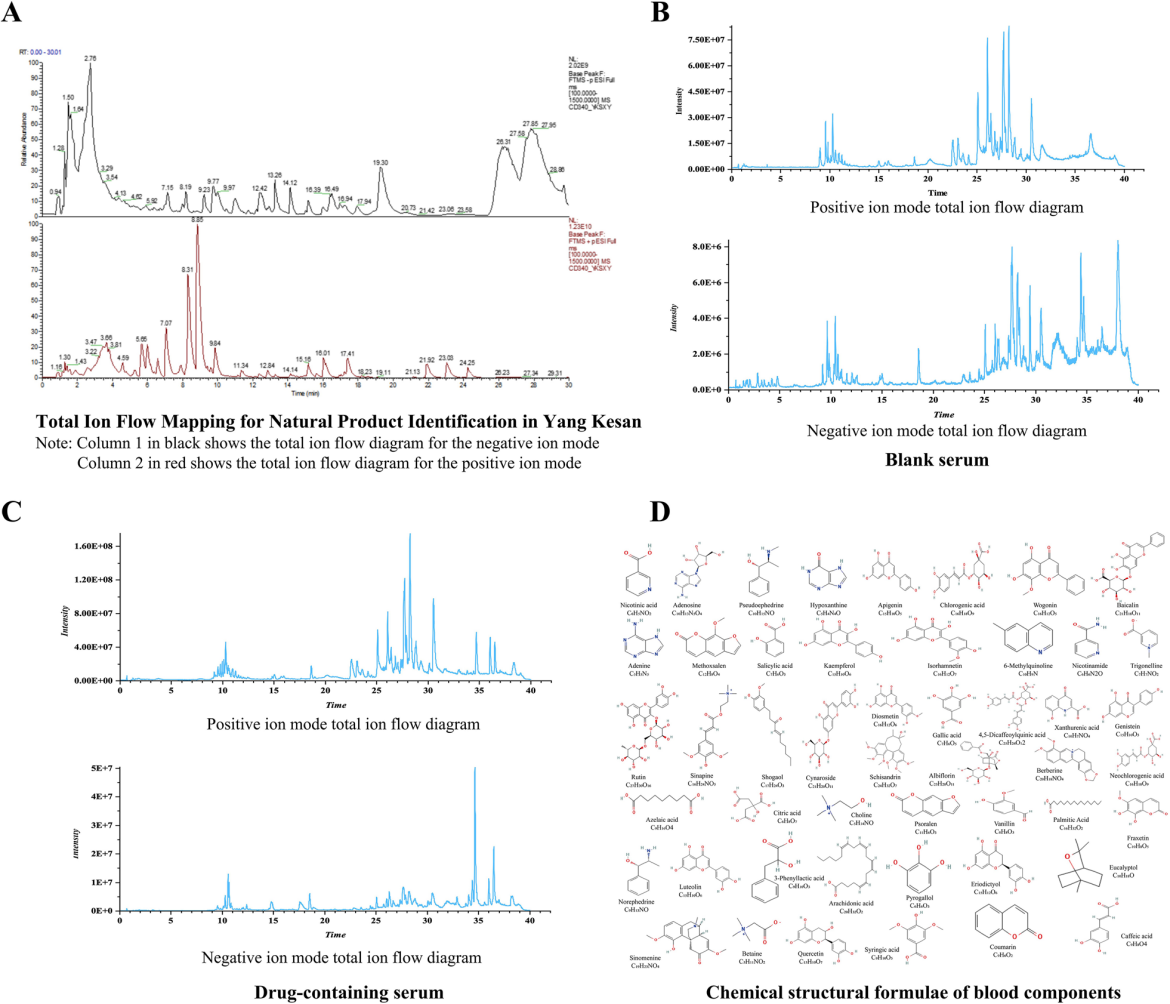
Analysis of the in vitro material basis and blood-absorbed components of YKS: **A** Total ionogram of the In Vitro Material Basis of YKS; **B** Total ionogram of Blank Serum; **C** Total ionogram of Drug-Containing Serum; **D** The 49 Blood-Absorbed Active Components of YKS

Using UPLC-Q-Exactive Orbitrap-MS technology, both positive and negative ion scans were applied to lyophilized YKS powder samples under previously optimized chromatographic and mass spectrometric conditions. This comprehensive analysis produced ion spectra in both modes (Fig. 2A), which were subsequently compared against the MzCloud database and our own literature database. Eventually, 174 distinct compounds were identified, comprising 39 flavonoids, 22 polyphenols, 10 triterpenoids, 19 alkaloids, 40 organic acids, 17 sugars and glycosides, 11 coumarins, and 16 other types of compounds. Depict the analytical results on both blank serum and serum from YKS-treated mice (Fig. 2B, C). Then, 49 components capable of entering the bloodstream (Fig. 2D) were successfully identified by integrating these results with the in vitro material composition of YKS. These blood-entry components included 12 flavonoids, 9 polyphenols, 1 triterpenoid, 10 alkaloids, 10 organic acids, 2 sugars and glycosides, 4 coumarins, and 1 additional compound. These compounds might explain the therapeutic effects of YKS on asthma, as detailed in Supplementary Tables S2 and S3.

### Establishment of network pharmacology for YKS blood components and allergic asthma

#### Identification of disease targets and collection of active blood components targets of YKS

**Fig. 3:**
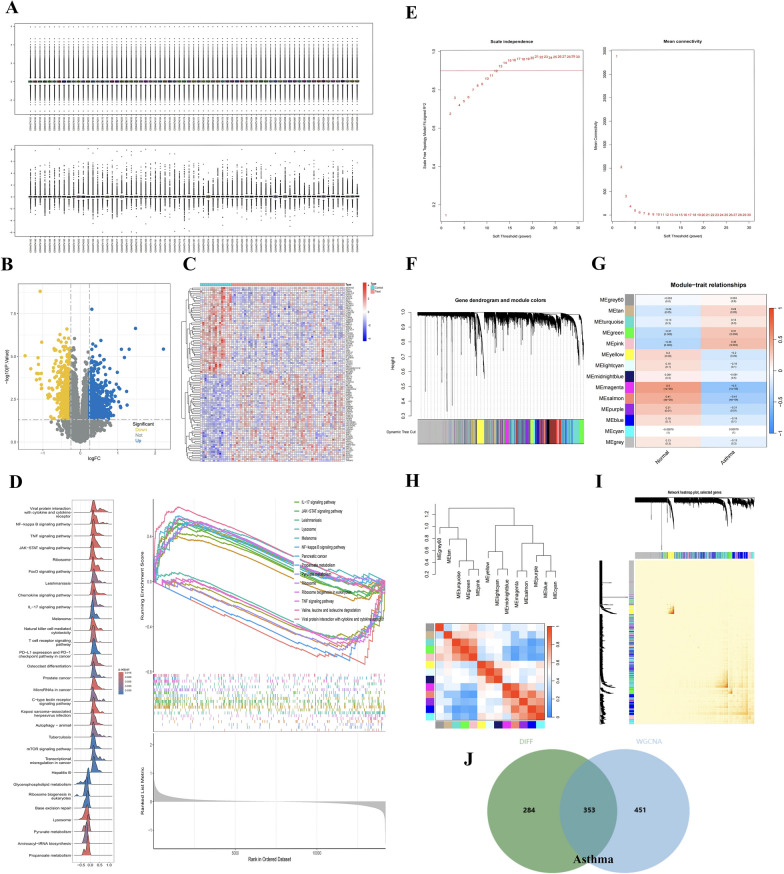
Prediction of asthma-associated targets. **A** Sample standardization of the GSE137268 dataset; **B**–**D** Differential analysis of the GSE137268 dataset, including differential gene volcano maps, differential gene heatmaps, and differential gene GSEA enrichment analysis; **E**–**I** WGCNA analysis of the GSE137268 dataset, including threshold selection, module visualization, correlations across modules and groups, module heatmaps, and module vs. trait heatmaps; **J** Intersection of differential analysis and WGCNA analysis for the identification of asthma-related targets

Analysis based on the GSE137268 dataset revealed differential expression of 637 genes, with 232 upregulated and 405 downregulated (Fig. 3A–C). GSEA identified significant enrichment of these genes in critical biological pathways, including NF-κB, TNF, JAK-STAT, IL-17, and T cell receptor signaling (Fig. 3D). A subsequent WGCNA applied to the same dataset, with a sample clustering threshold of 12, led to the identification of 12 modules that were integral in forming a co-expression network. Notably, two modules of salmon (r = 0.50, P = 1e − 05) and magenta (r = 0.41, P = 4e − 04), containing 804 genes, demonstrated strong associations with asthma (Fig. 3E-I). Integration of these 804 genes from the WGCNA analysis with the 637 DEGs previously linked to asthma facilitated the delineation of 353 potential asthma-relevant targets (Fig. 3J). To assess the effects of 49 blood-active components, further exploration using the STP method found that 972 targets were identified to be potentially relevant to the therapeutic properties of YKS.

#### Potential therapeutic targets of YKS for asthma, PPI, enrichment analysis, and network diagram construction

**Fig. 4:**
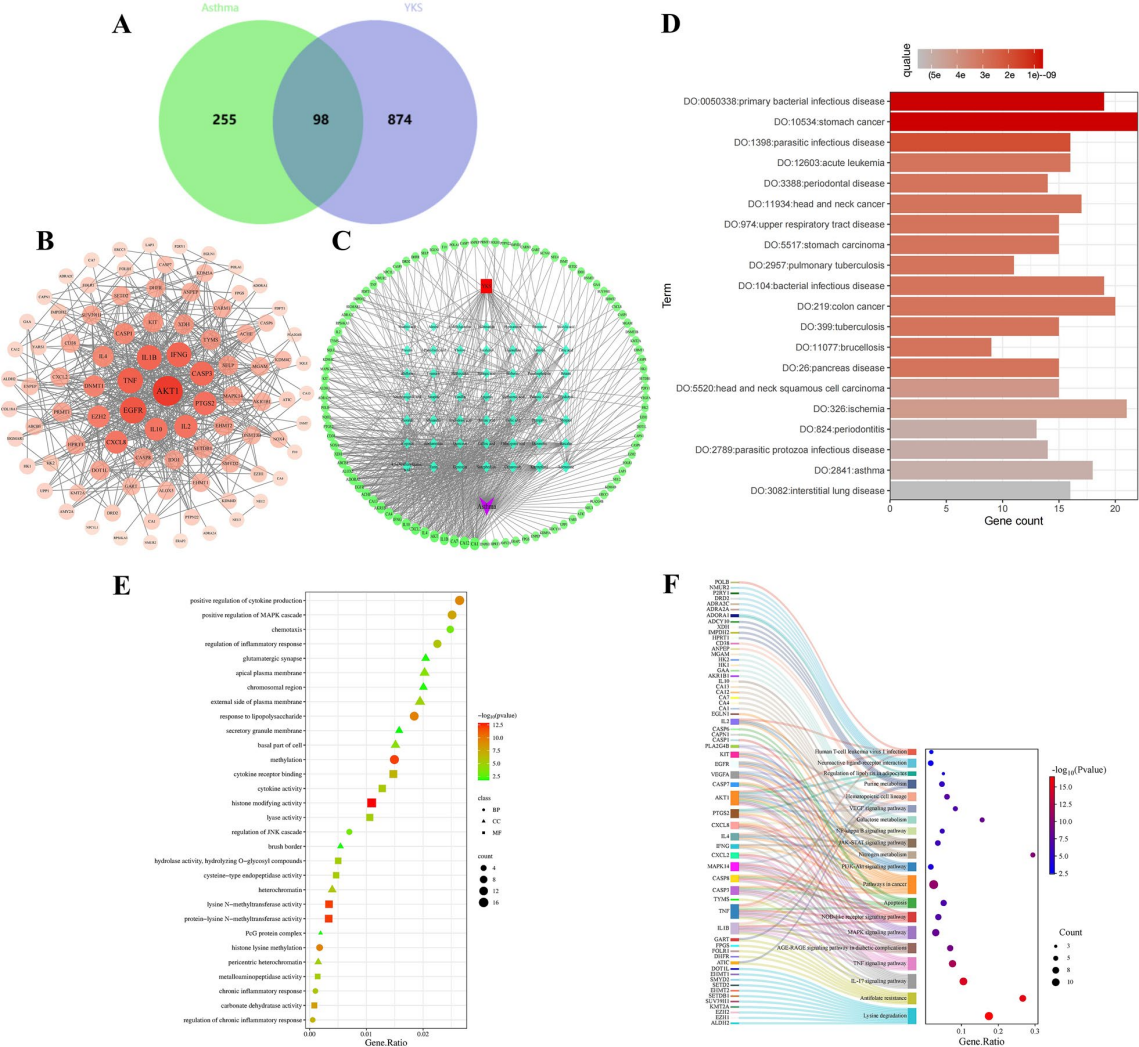
Mechanism prediction of YKS in treating asthma: **A** Core targets of YKS in asthma treatment; **B** PPI network diagram; **C** Construction of the network diagram; **D** DO enrichment analysis; **E** GO enrichment analysis; **F** KEGG enrichment analysis

In this study, we employed R to identify 98 targets that overlap between those influenced by the blood-active components of YKS and those associated with asthma, underscoring their potential utility in YKS-based asthma therapy (Fig. 4A). Then, a PPI network, visualized using Cytoscape, was constructed by uploading these targets into the STRING database. This analysis facilitated the identification of principal targets by evaluating their correlation coefficients, pinpointing AKT1, TNF, IL1B, EGFR, IFNG, IL4, CASP3, and PTGS2, which might be critical in explaining the potential efficacy of YKS for asthma treatment (Fig. 4B). Furthermore, a network diagram distinguishing YKS blood components (in cyan) from the corresponding potential therapeutic asthma targets (in green) was plotted by integrating these core targets into Cytoscape. It was identified that Kaempferol, Norephedrine, Cynaroside, Genistein, and Rutin were core active constituents of YKS, potentially forming the foundational elements for its effectiveness in asthma management (Fig. 4C). These findings are detailed in Supplementary Table S4.

Through DO enrichment analysis, 98 YKS targets implicated in asthma treatment revealed significant associations, underscored their relevance to respiratory health conditions such as tuberculosis, primary bacterial infections, upper respiratory tract diseases, and asthma itself (Fig. 4D). This confirmation of the relevance of these YKS targets to respiratory health is detailed in Supplementary Table S5. GO enrichment in the Biological Processes (BP) category highlighted activities such as the positive regulation of cytokine production, enhancement of MAPK cascade signaling, modulation of inflammatory responses, response to lipopolysaccharide, and control of chronic inflammation (Supplementary Table S6). For the Cellular Components (CC) category, notable enrichment included structures such as glutamatergic synapses, the external side of the plasma membrane, and the basal cellular region (Supplementary Table S7). Molecular Function (MF) enrichment analysis identified critical roles in cytokine receptor binding, cytokine activity, metalloaminopeptidase activity, and carbonate dehydratase activity (Fig. 4E), detailed in Supplementary Table S8. Further analysis of KEGG pathways illuminated crucial pathways involved in the therapeutic effects of YKS. These include the PI3 K-Akt signaling pathway, NF-kappa B signaling pathway, VEGF signaling pathway, apoptosis, NOD-like receptor signaling pathway, MAPK signaling pathway, TNF signaling pathway, and IL-17 signaling pathway. The key targets involved in these pathways are depicted in Fig. 4F and detailed in Supplementary Table S9.

#### Molecular docking and Mendelian randomization analysis

**Fig. 5:**
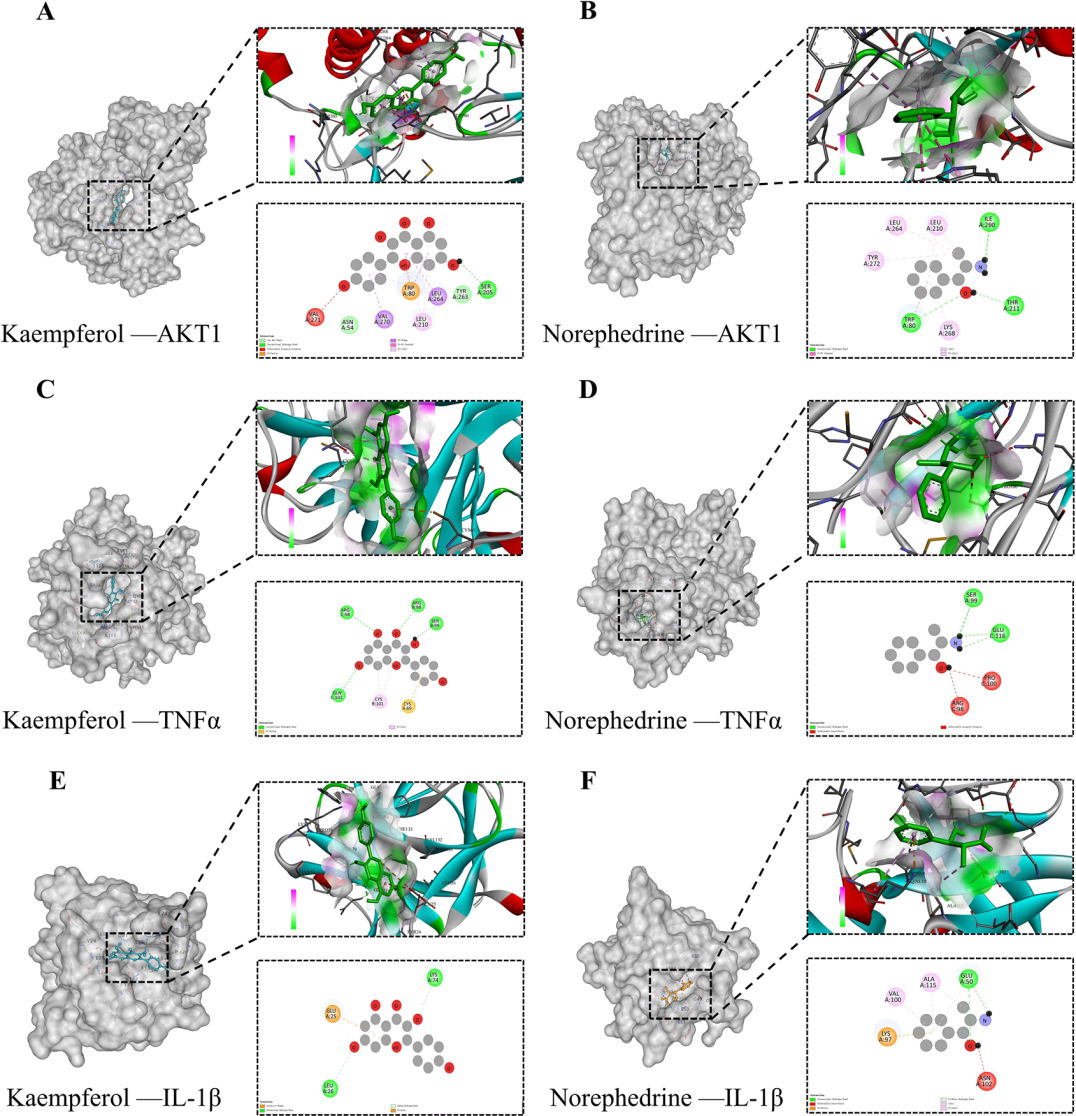
Molecular docking diagram. Results are shown as 3D and 2D plots. **A** Molecular docking model of Kaempferol with AKT1. **B** Norephedrine with AKT1 molecular docking model. **C** Docking model of Kaempferol with TNFα molecule. **D** Norephedrine with TNFα molecular docking model. **E** Kaempferol with IL-1β molecular docking model. **F** Norephedrine with IL-1β molecular docking model

Based on the aforementioned network analysis, Kaempferol and Norephedrine were the principal active compounds in YKS for treating asthma. These components were subjected to molecular docking studies to evaluate their interaction with key asthma-related targets such as AKT1, TNF, and IL1B. The results indicated lower binding energies, signifying enhanced binding capabilities and suggesting a strong affinity between these YKS components and the identified targets (Fig. 5A–F and Supplementary Fig.S7). MR analysis revealed a significant causal link between AKT1 and asthma, evidenced by an odds ratio (OR) of 1.097 with a 95% confidence interval (CI) of 1.019–1.181 and a P-value of 0.013. In contrast, no causal associations were found for TNF (OR = 1.024, 95% CI 0.962–1.091; P = 0.445) and IL1B (OR = 0.990, 95% CI 0.934–1.049; P = 0.741). Tests for pleiotropy and heterogeneity in the association between AKT1 and asthma yielded results greater than 0.05, reinforcing the robustness of these findings. This was further validated by a leave-one-out sensitivity analysis, with detailed results depicted in Supplementary Fig.S1–6.

#### YKS reduces airway resistance changes in mice

**Fig. 6:**
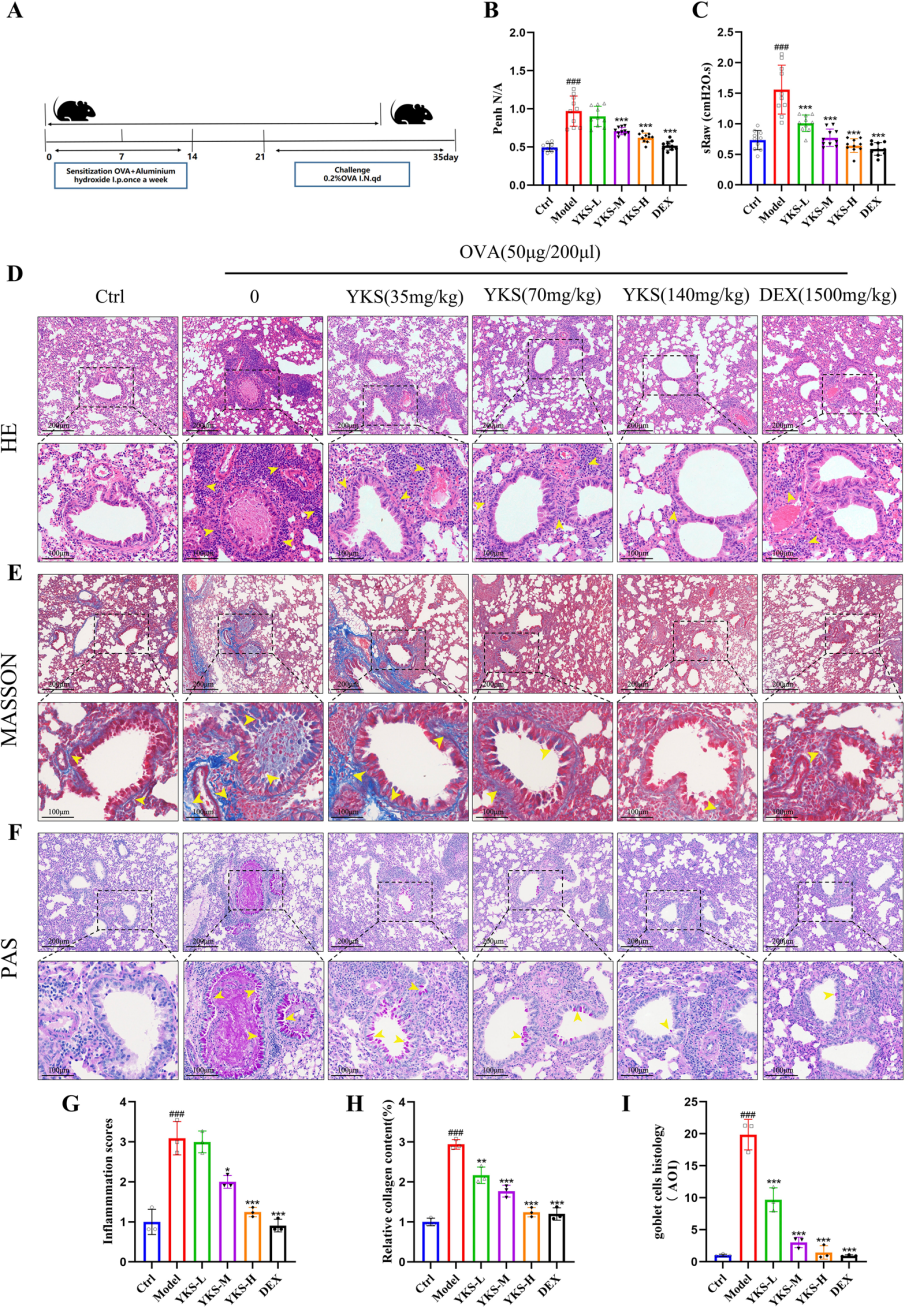
YKS mitigates pulmonary dysfunction, airway inflammation, remodeling, and excessive mucus production in an ovalbumin-induced asthma model in mice: **A** Schematic depiction of the OVA-induced asthma model in mice; **B** Evaluation of bronchial constriction through the PenH index in mice; **C** Measurement of airway resistance in mice, indicated as sRAW; **D** Histological evaluation of lung tissues with Hematoxylin and Eosin stain at 200× magnification; **E** Analysis of lung tissues using Masson’s trichrome staining technique at 200× magnification; **F** Detection of mucus secretion in lung tissues using Periodic Acid-Schiff stain at 200 × magnification; **G**–**I** Detailed quantitative histopathological assessments of lung tissues. Data are presented as mean ± standard deviation, with three samples per group. Statistical significance relative to the blank group is indicated as follows: ^#^P < 0.05, ^##^P < 0.01, ^###^P < 0.001; significance relative to the control group is denoted by *P < 0.05, **P < 0.01, ***P < 0.001

In this study, pulmonary functions were evaluated using standardized tests across different groups. The control group exhibited typical airway resistance levels, indicative of standard pulmonary function. On the contrary, mice in the OVA group demonstrated significantly increased airway resistance, signifying compromised pulmonary function. Remarkably, treatment with both YKS and DEX led to substantial reductions in airway resistance in OVA-induced asthmatic mice. This improvement was revealed in a notable decrease in the PenH index, indicating reduced airway constriction. The effectiveness of these treatments in mitigating airway resistance is documented in Fig. 6B, C.

#### YKS alleviates airway inflammation, excessive mucus secretion, and airway remodeling induced by OVA exposure in mice

Histological examination of pulmonary sections from the experimental asthma cohort via H&E staining revealed marked increases in inflammatory cell infiltration around both airways and blood vessels. Comparative analysis indicated significantly reduced infiltration levels in mice after YKS treatment, as detailed in Fig. 6D and G. Furthermore, PAS staining was employed to quantify the capacity of YKS to curb OVA-induced mucus production by epithelial cells and the proliferation of goblet cells. PAS-positive cells were substantially more prevalent in asthmatic mice compared to the control group. However, a significant reduction in these cells was observed after YKS treatment (Fig. 6F and I). In addition, Masson’s Trichrome staining was utilized to evaluate the accumulation of collagen and fibrous materials in the bronchial pathways. It was observed with a pronounced decrease in such accumulations after YKS treatment, compared to their asthmatic counterparts (Fig. 6E and H).

#### YKS inhibits OVA-induced expression of Th2 cytokines in BALF and serum immunoglobulin E levels

**Fig. 7:**
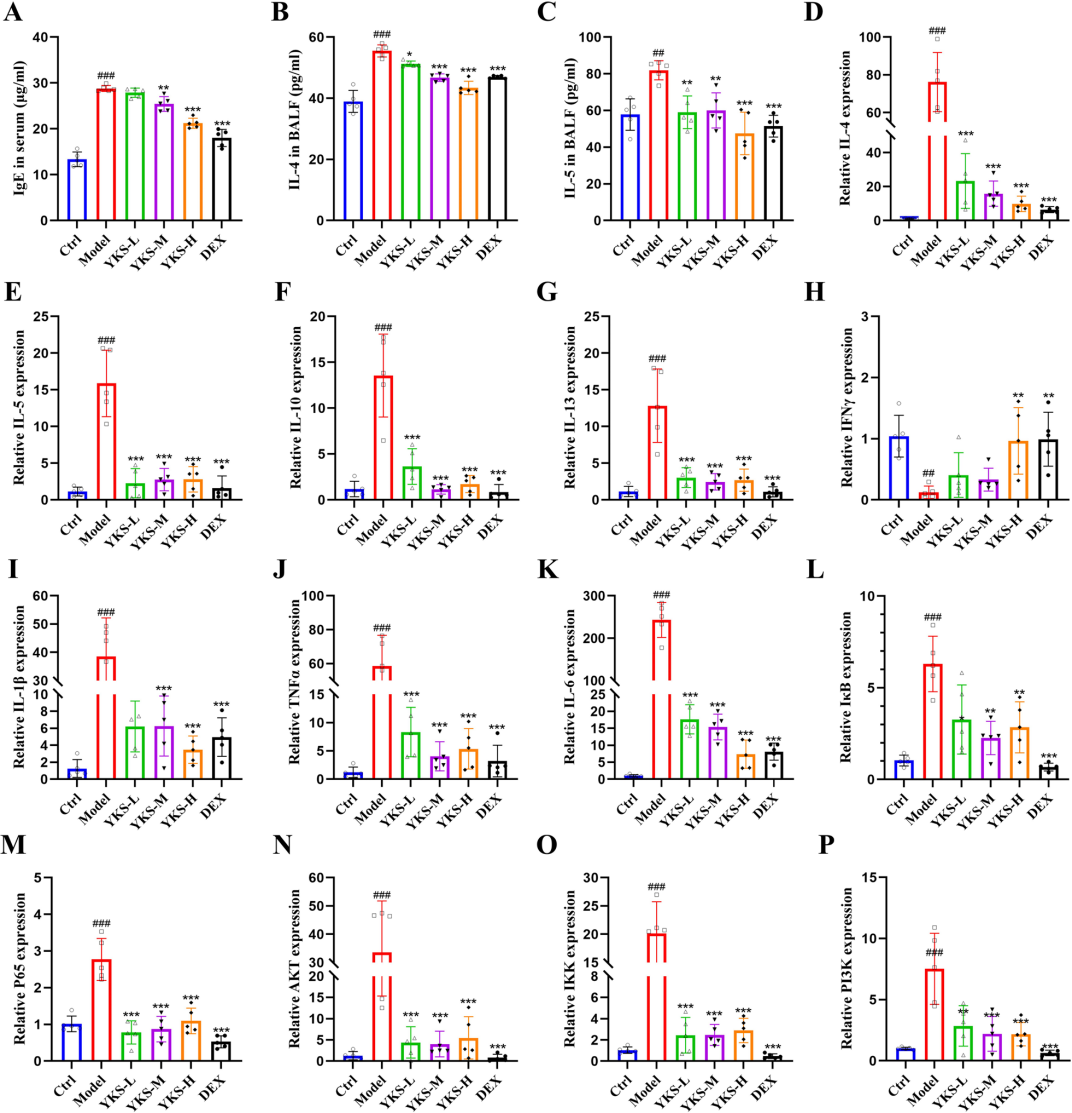
YKS’s influence on asthma-related markers in mice: this figure illustrates the effectiveness of YKS in reducing key inflammatory and signaling molecules in asthma-induced mice. **A** Utilization of ELISA to measure total serum IgE levels; **B**, **C** Determination of IL-4 and IL-5 in BALF via ELISA; **D**–**G** Real-time PCR analyses reveal the mRNA expressions of IL-4, IL-5, IL-10, and IL-13 in lung tissues, respectively; **H** IFNγ mRNA levels assessed by qPCR in lung tissue; **I**–**K** Quantification of pro-inflammatory cytokines IL-1β, TNFα, and IL-6 mRNA in lung tissues via qPCR; **L**, **M** Expression levels of NF-κB pathway components IκBα and P65 evaluated through qPCR; **N**, **O** Assessment of AKT and IKK mRNA expressions in lung tissues; **P** Measurement of PI3K mRNA levels using qPCR. All data are represented as mean ± SD for five specimens per group. Statistical significance is denoted as follows: compared with the blank group, ^#^P < 0.05, ^##^P < 0.01, ^###^P < 0.001; compared with the control group, *P < 0.05, **P < 0.01, ***P < 0.001

Existing evidence has documented a link between the onset of allergic asthma and an increase in the secretion of type 2 cytokines. In this context, this study detected the concentration of Th2 cytokines in BALF using ELISA. Sensitized mice that had been exposed to OVA exhibited significant increases in IL-4 and IL-5 levels, accompanied by a considerable rise in serum IgE levels concurrently. Following intervention with YKS and DEX, these elevated levels were effectively attenuated, showing a pronounced reduction in the expressions of IgE, IL-4, and IL-5 (Fig. 7A–C). Therefore, YKS may be particularly effective in substantially reducing cytokine concentrations in both BALF and serum, thereby contributing significantly to the mitigation of asthmatic responses.

#### YKS suppresses mRNA expression of inflammatory cytokines in lung tissue

To assess the regulatory effect of YKS on the Th2-mediated immune response in asthma, lung tissue samples from the study cohorts were analyzed for Th2 cytokine gene expression using qPCR. Mice sensitized and then exposed to OVA exhibited a pronounced increase in IL-4, IL-5, IL-10, and IL-13 levels (Fig. 7D–G). Conversely, the expression of the anti-inflammatory cytokine IFN-γ was considerably decreased (Fig. 7H). Notably, both YKS and DEX interventions resulted in significant suppression of Th2 cytokine expression, along with an upregulation of IFN-γ, indicating effective modulation of the allergic inflammatory response. Additionally, as illustrated in Fig. 7I–K, the OVA-induced model also recorded heightened leukocyte infiltration and increased levels of pro-inflammatory cytokines such as TNF-α, IL-6, and IL-1β. Following the administration of YKS and DEX, there was a notable reduction in the concentrations of these inflammatory markers. Collectively, YKS may function as a therapeutic agent, enabling effective diminution of Th2 dominance in the immune responses characteristic of allergic asthma, suggesting its utility in managing this condition.

#### YKS inhibits activation of the PI3 K/AKT/NF-κB signaling pathway in OVA-induced asthmatic mice

**Fig. 8:**
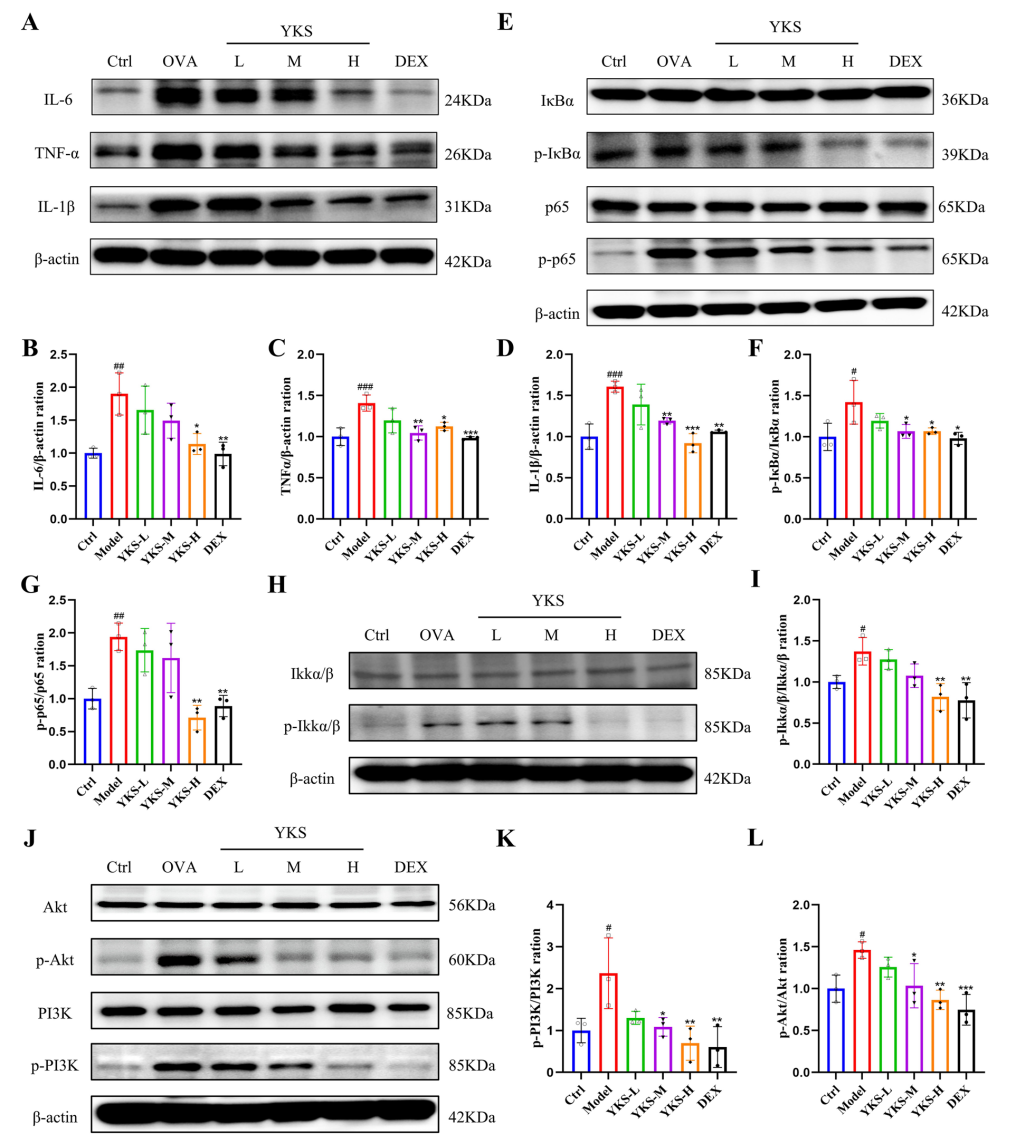
YKS Modulation of the PI3 K/AKT/NF-κB Signaling pathway in OVA-challenged asthma model mice: **A** Western blot analysis reveals protein levels of IL-6, TNFα, IL-1β, and β-actin as internal control; **B**–**D** Densitometric analysis of the bands was performed to quantify the relative protein levels of IL-6, TNFα, and IL-1β, normalized to β-actin; **E** Detection of phosphorylated and total IκBα, P65 via Western blot, with β-actin as a loading control; **F**–**G** Quantitative analysis of p-IκBα and p-P65; **H** Western blot identification of phosphorylated and total IKK, with β-actin serving as the control; **I** Quantitative analysis of IKK phosphorylation levels; **J** Western blotting analysis of phosphorylated and total forms of PI3 K and AKT, with β-actin as a loading control; **K**–**L** Relative phosphorylation levels of PI3K and AKT are quantified. All values are expressed as mean ± SD for three samples per group. Significance of differences: ^#^P < 0.05, ^##^P < 0.01, ^###^P < 0.001 when compared with the blank group; *P < 0.05, **P < 0.01, ***P < 0.001 relative to the control group

**Fig. 9:**
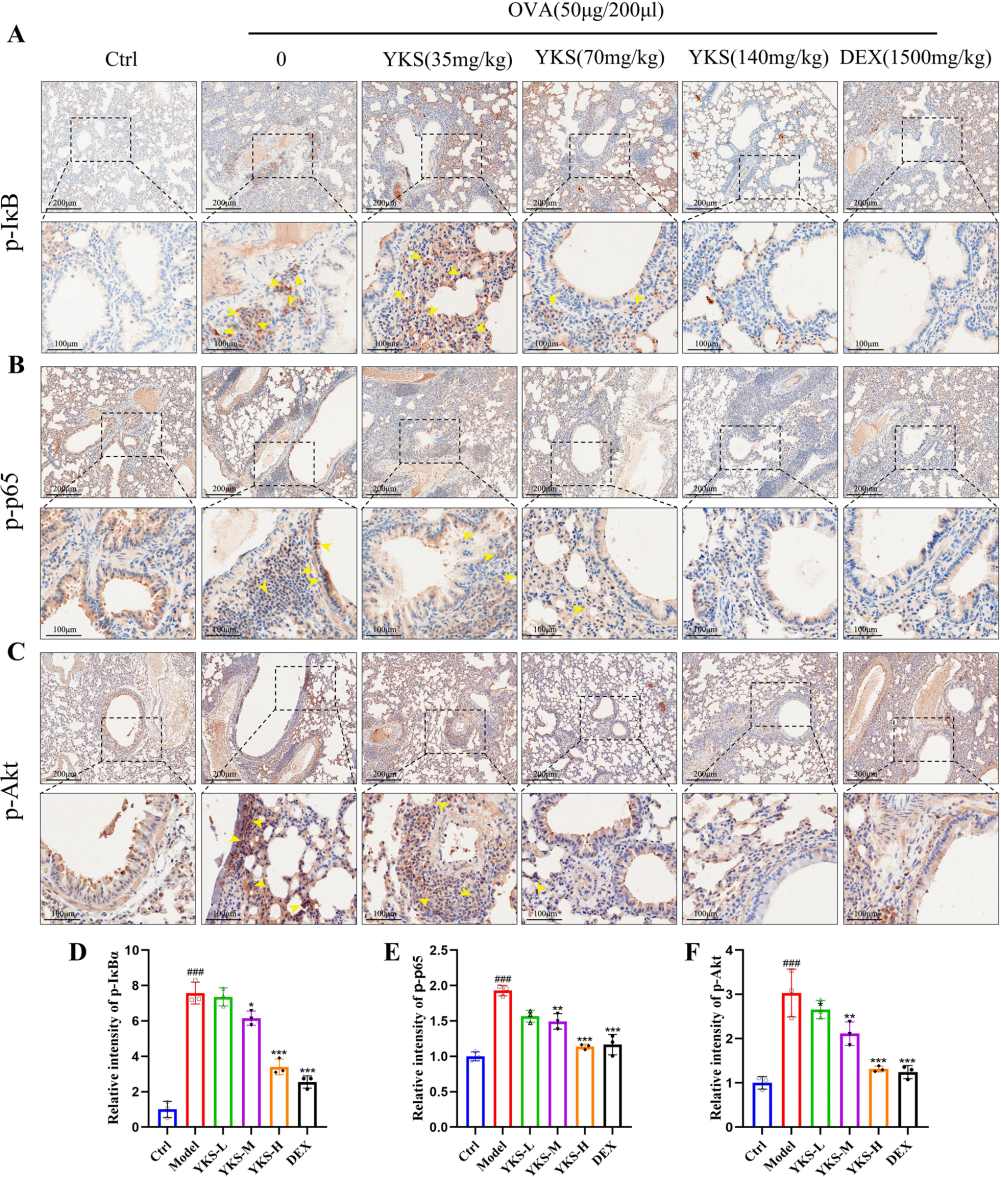
Modulation of Phosphorylated Signaling Proteins by YKS in Lung Tissues of Asthmatic Mice: **A**–**C** Immunohistochemical analysis was performed to detect the levels of phosphorylated IκBα, P65, and AKT in lung tissues from mice in the blank, OVA, YKS, and DEX groups, with images captured at 200 × magnification; **D**–**F** Quantification of the immunohistochemical staining was achieved using ImageJ software, assessing the expression levels of these phosphorylated proteins. Statistical analysis shows that the values are presented as mean ± SD, with three animals per group. Relative to the blank group, changes in phosphorylation are significant, with ^#^P < 0.05, ^##^P < 0.01, ^###^P < 0.001; and in comparison to the control group, significant alterations are noted at *P < 0.05, **P < 0.01, ***P < 0.001

YKS might mitigate allergic airway inflammation in asthma by modulating the PI3 K/AKT/NF-κB signaling pathway, according to previous integrated analyses using bioinformatics, network pharmacology, and MR. To validate this hypothesis, qPCR was utilized and detected an upregulation of PI3 K, AKT, IKK, IκB, and P65 in mice exposed to OVA. Notably, these levels were attenuated following treatment with YKS and DEX (Fig. 7L–P). Research highlights the pivotal role of the NF-κB pathway among various intracellular pathways influencing T cell differentiation. Following OVA administration, IL-6, IL-1β, and TNFα levels were elevated (Fig. 8A–D), which in turn activated the NF-κB pathway, as evidenced by enhanced phosphorylation of key proteins in the PI3 K/AKT/NF-κB cascade. Noticeably, as shown in Fig. 8E–L, YKS effectively inhibited this phosphorylation, reducing activity levels of key pathway components. Consequently, it can be speculated that YKS curtails OVA-induced allergic airway inflammation by suppressing the PI3 K/AKT/NF-κB cascade. Additional immunohistochemical analyses revealed elevated phosphorylation of AKT, P65, and IκB in lung tissues post-OVA challenge. This phosphorylation was significantly diminished by YKS treatment, as depicted in Fig. 9A–F.

## Discussion

Asthma is a prevalent chronic inflammatory condition of the airways that is increasingly recognized in China. Despite its growing recognition, it often remains undiagnosed and is frequently inadequately treated [23]. Both environmental and genetic factors play crucial roles in the onset and progression of asthma [24, 25], leading to diverse pathological alterations in lung tissue. These alterations compromise respiratory efficiency and significantly degrade the quality of life [26]. Moreover, long-term use of pharmacological agents is known to potentially trigger significant adverse effects in asthmatic patients [27, 28]. Our previous studies have demonstrated that YKS not only mitigates asthma symptoms but also significantly improves the quality of life and reduces the incidence of acute exacerbations in asthma patients. However, there is so far insufficient data to uncover the in vitro and in vivo components as well as the exact mechanisms by which YKS exerts its effects. In this research, using UPLC-Q-Exactive Orbitrap-MS, we delineated 174 in vitro components and 49 in vivo components that were absorbed into the bloodstream from YKS. This study continued to investigate the mechanism underlying the effect of YKS alleviating allergic airway inflammation in asthma using advanced methods such as network pharmacology, molecular docking, MR, and controlled animal studies. The findings reveal that YKS effectively reduces airway resistance, moderates pathological changes and inflammatory cell infiltration in lung tissues, lowers cytokine levels in BALF and serum, and adjusts the activation of PI3 K/AKT/NF-κB signaling pathway.

The imbalance between Th2 and Th1 lymphocytes represents a predominant etiology of asthma [29], leading to over-production of Th2 cytokines (IL-4, IL-5, IL-13) and a decrease in IFN-γ, driving the pathophysiological changes in asthma [30]. This imbalance can promote eosinophil, lymphocyte, and mast cell infiltration into the airways, together with increased mucus production and subepithelial fibrosis, ultimately leading to the airway narrowing characteristic of asthma-related structural changes in bronchial and lung tissues [31, 32]. Th2 cells, influenced by IL-4, promote the maturation of B cells and subsequent IgE secretion by plasma cells. This IgE binds to mast cell surfaces, triggering the release of active mediators that exacerbate asthma symptoms [33–35]. Moreover, IgE and IL-5 jointly amplify eosinophil recruitment and activation, and also promote neutrophil aggregation. Similarly, IL-13, a cytokine with multiple roles, can support B cell proliferation and antibody production, and enhance IgE synthesis, driving persistent airway inflammation. IL-13 may also induce goblet cell metaplasia and increase Muc5ac expression in airway epithelia, leading to excessive mucus production, contributing to airway hyperreactivity and remodeling [36–38]. Altogether, these cytokines serve as both biomarkers and therapeutic targets for asthma [39, 40]. Our in vivo animal experiments verified the effects of YKS on improving airway resistance, as well as reducing inflammation, mucus secretion, and airway remodeling in asthmatic mice. It effectively decreased the levels of Th2 cytokines and IgE in the serum and BALF. Furthermore, qPCR analyses confirmed that YKS significantly reduced the expression of Th2 cytokines (IL-4, IL-5, IL-10, IL-13) and increased that of IFN-γ, while also diminishing the levels of pro-inflammatory cytokines (TNF-α, IL-6, IL-1β). Consequently, YKS may alleviate asthma-associated inflammatory processes by correcting the Th2/Th1 imbalance.

Furthermore, this study utilized network pharmacology to identify pivotal asthma treatment targets for YKS, including AKT1, TNF, IL1B, EGFR, IFNG, IL4, CASP3, and PTGS2. AKT1, downstream of PI3 K, can regulate various growth factors impacting lung function in asthma [41], modulate inflammatory cytokine IL6 [42], and inhibit proliferation in human bronchial smooth muscle cells [43]. Interestingly, AKT1 is also noted as a potential therapeutic target in COVID-19 management [44]. TNF, a versatile pro-inflammatory cytokine, influences cellular proliferation, differentiation, and apoptosis, playing a significant role in the pathogenesis of asthma [45]. Serving as a foundational element in asthma therapy [46], TNF can interact with TNF-like ligands and IL33 to trigger allergic airway inflammation [47], which has also been regarded as a marker for allergic pulmonary disorders [48]. Furthermore, it sustains chronic type 2 airway inflammation, making it a critical target for asthma treatment [49]. IL1B, essential for mediating inflammatory responses, promotes neutrophil recruitment to epithelial cells [50] and is linked to allergen sensitization and the development of allergic asthma [51]. Moreover, IFNG is critical for regulating asthma airway hyperreactivity [52], and EGFR can affect distal airway epithelial responses to allergies [53] and manage airway epithelial inflammation [54]. Mendelian randomization was employed to investigate causal relationships between asthma and core targets like AKT1, TNF, and IL1B, revealing a direct causality with AKT1 but not with TNF or IL1B, suggesting their indirect influence on asthma pathways. In terms of the key active components of YKS, Kaempferol is known for its anti-inflammatory and antioxidant effects [55], which works to protect against acute lung injury [56], manages oxidative stress [57], and enhances handling of allergic responses [58], playing a role in energy damage response [59]. Norephedrine affects bronchial smooth muscle contraction [60], while Genistein exhibits anti-inflammatory properties [61], reducing the frequency of acute asthma episodes [62]. Our molecular docking confirmed interactions of these active components, particularly Kaempferol and Norephedrine, with core targets AKT1, TNF, and IL1B, underscoring their potential therapeutic roles in managing asthma.

**Fig. 10:**
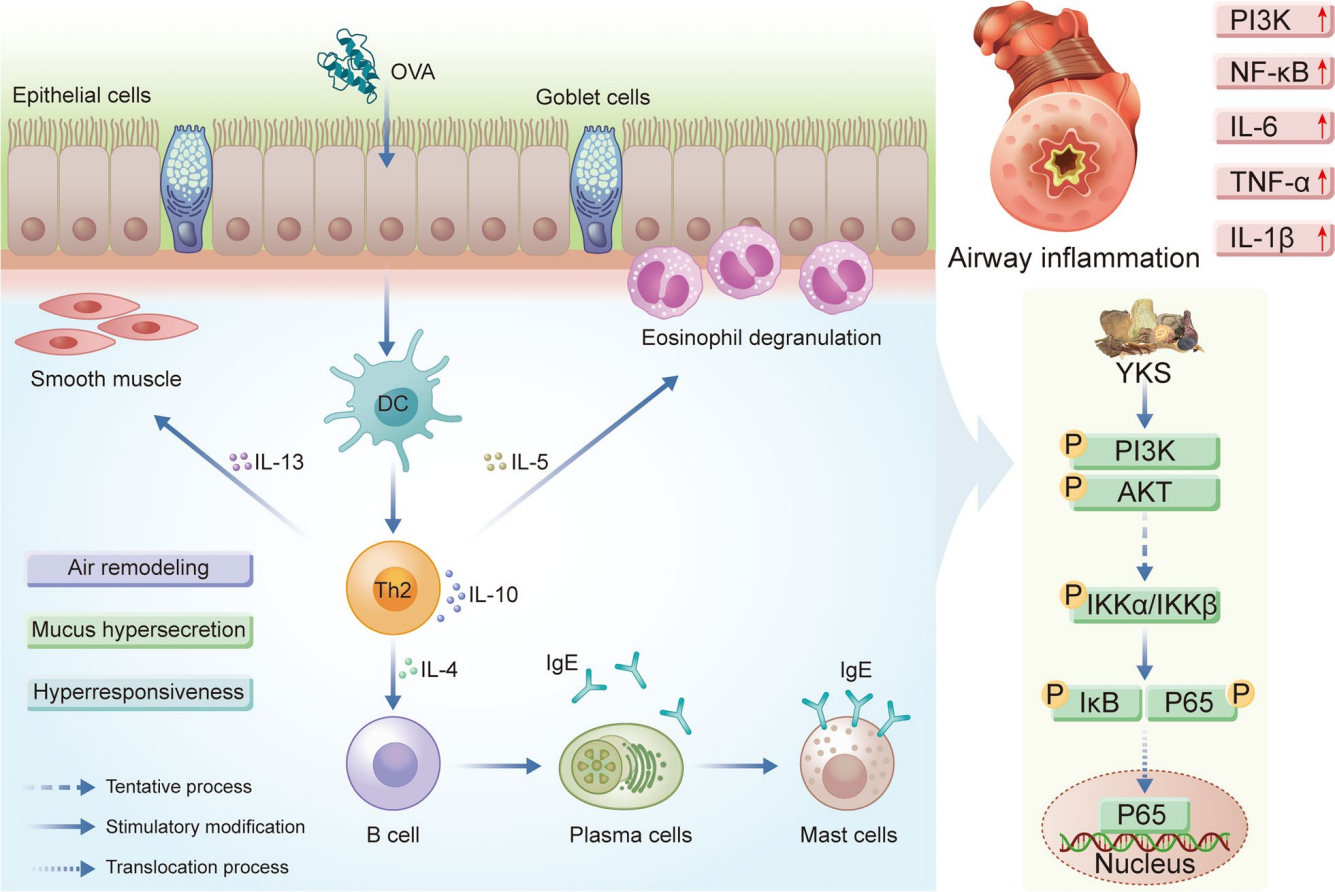
Molecular mechanisms of YKS for asthma

Enrichment analyses of asthma-relevant YKS targets identified DO terms significantly associated with respiratory diseases, most notably asthma, along with tuberculosis, primary bacterial infections, and upper respiratory tract disorders. Therefore, YKS may exert effects on asthma progression through these identified targets. GO enrichment findings further suggest that YKS's therapeutic action in asthma may involve enhancing cytokine production, modulating the inflammatory response, and particularly targeting chronic inflammation, which could help in alleviating the symptoms of asthma. Pathway analysis via KEGG underscored the significance of the PI3K-Akt and NF-kappa B signaling pathways in this context. Both the PI3K/AKT pathway and its key downstream mediator NF-κB are critical in controlling cell growth, proliferation, and survival through the regulation of various downstream elements [63, 64]. NF-κB can control the expression of genes related to inflammation in airway cells [65, 66], with its activation prompted by cytokines like IL-6, IL-1β, TNFα, and triggered by Toll-like receptors in various airway cell types, subsequently influencing the expression of immune and inflammatory mediators [67, 68]. Upon stimulation by inflammatory mediators, NF-κB proteins in the cytoplasm are activated and translocated into the nucleus to modulate various inflammatory factors, crucially impacting allergic responses [69, 70]. The PI3K/AKT signaling pathway involves the interaction of the lipid kinase family PI3K with the PH domain of AKT, leading to the activation of AKT, which then moves from the cytoplasm to the plasma membrane, activating downstream effectors. Inhibitors of PI3K are thus considered potent anti-inflammatory agents [71], and the pathway regulates the differentiation of T helper cells and eosinophils in asthma, impacting the progression and severity of inflammatory responses in asthma [72]. Our investigations based on immunohistochemistry, qPCR, and western blot further supported the effective role of YKS in modulating the mechanism of allergic airway inflammation in asthma by inhibiting the PI3K/AKT/NF-κB signaling pathway. In a word, findings in this study may lay a theoretical foundation for the potential utilization of YKS in asthma therapy, as visualized in Fig. 10.

## Conclusion

This study identifies the in vitro chemical components and the bioavailable constituents of YKS absorbed into the bloodstream by employing UPLC-Q-Exactive Orbitrap-MS technology. Furthermore, this study screens active substances such as Kaempferol, Norephedrine, Cynaroside, Genistein, and Rutin, which can target critical proteins including AKT1, TNF, IL1B, EGFR, IFNG, IL4, CASP3, and PTGS2, to function significantly in the pathogenesis of asthma. The modulation of the PI3K/AKT/NF-κB signaling pathway by these constituents can effectively alleviate allergic airway inflammation, thus contributing to their therapeutic impact on asthma. Findings in this study may lay a theoretical groundwork for advancing the application of YKS in asthma management.

## Supplementary Information

Additional file 1

Additional file 2

Additional file 3

Additional file 4

Additional file 5

Additional file 6

Additional file 7

Additional file 8

Additional file 9

Additional file 10

## Data Availability

The datasets used and/or investigated during the current study are available from the corresponding author upon reasonable request.

## Abbreviations

YKS: Yangke Powder (yǎng ké sǎn)
TCM: Traditional Chinese Medicine
UPLC: Ultra-performance liquid chromatography
OVA: Ovalbumin
HE: Hematoxylin and eosin
PAS: Periodic acid-Schiff
ELISA: Enzyme-linked immunosorbent assay
qPCR: Quantitative real-time polymerase chain reaction
IgE: Immunoglobulin E
IL-4: Interleukin-4
IL-5: Interleukin-5
IL-6: Interleukin-6
IL-10: Interleukin-10
IL-13: Interleukin-13
IFN-γ: Interferon-gamma
IL-17: Interleukin-17
IL-33: Interleukin-33
Th1: T helper 1 cell
Th2: T helper 2 cell
AKT1: Serine/Threonine-Protein Kinase 1
TNF: Tumor necrosis factor
IL1B: Interleukin-1 beta
EGFR: Epidermal growth factor receptor
IFNG: Interferon gamma
CASP3: Cysteine-containing aspartate-specific protease 3
PTGS2: Prostaglandin-endoperoxide synthase 2
PI3K: Phosphatidylinositol 3-kinases
NF-κB: Nuclear factor kappa-light-chain-enhancer of activated B cells
IKKα/β: Inhibitor of nuclear factor kappa-B kinase subunit alpha/beta
IκB: Inhibitor of nuclear factor kappa-B
MAPK: Mitogen-activated protein kinase
VEGF: Vascular endothelial growth factor
NOD: Nucleotide-binding oligomerization domain
BW: Body weight
MR: Mendelian randomization
No: Number
SPF: Specific pathogen-free
SD: Sprague-dawley
ESI: Electrospray ionization
NCE: Normalized collision energy
TCMSP: Traditional chinese medicine systems pharmacology database and analysis platform
PPM: parts per million
GEO: Gene expression omnibus
DEGs: Differentially expressed genes
GSEA: Gene set enrichment analysis
WGCNA: Weighted gene co-expression network analysis
STP: Swiss target prediction platform
SDF: Structure data file
SMILES: Simplified molecular input line entry system
PPI: Protein-protein interaction
DO: Disease ontology
GO: Gene ontology
KEGG: Kyoto encyclopedia of genes and genomes
BP: Biological processes
CC: Cellular components
MF: Molecular function
GWAS: Genome-wide association study
SNP: Single-nucleotide polymorphisms
IVW: Inverse-variance weighted
DEX: Dexamethasone
NAM: Non-invasive airway monitoring system
sRaw: Specific airway resistance
WBP: Whole-body plethysmography system
PenH: Enhanced pause
BALF: Bronchoalveolar lavage fluid
DAB: 3,3'-Diaminobenzidine
BCA: Bicinchoninic acid
SDS-PAGE: Sodium dodecyl sulfate - polyacrylamide gel electrophoresis
PVDF: Polyvinylidene fluoride
BSA: Bovine serum albumin
