# Supplementary material for: Yangke powder alleviates OVA-induced allergic asthma by inhibiting the PI3K/AKT/NF-κB signaling pathway
Source: Chin Med. 2025 May 26;20:69. doi: 10.1186/s13020-025-01125-x (PMC12105270; doi:10.1186/s13020-025-01125-x)
Supplement: Supplementary file 1 — Additional file 1 [file 13020_2025_1125_MOESM1_ESM.docx]

**Yangke Powder Alleviates OVA-Induced Allergic Asthma by Inhibiting the PI3K/AKT/NF-κB Signaling Pathway**

**Xueyan Li^1, †^, Lu Ding^2, 1, †^, Zirui Li^1^, Zhenghua Cao^3^, Min Li^1^, Kai Yin^1^，Siyu Song^1^, Liyuan Cao^1^,Qinjing Xia^1^, Zihan Wang^1^, Daqing Zhao^1^, Xiangyan Li^1*^, Xiaolin Tong^1, 4*^ Zeyu Wang^1, 2*^**

^1^ Northeast Asia Research Institute of Traditional Chinese Medicine, Changchun University of Chinese Medicine, Changchun 130021, China.

^2^Research Center of Traditional Chinese Medicine, The Affiliated Hospital to Changchun University, Changchun, Jilin 130021, China.

^3^College of Traditional Chinese Medicine, Changchun University of Chinese Medicine, Changchun Jilin , China

^4^Institute of Metabolic Diseases, Guang'anmen Hospital, China Academy of Chinese Medical Sciences, Beijing 100053, China.

^†^Xueyan Li and Lu Ding contributed equally to this work.

***Correspondence:**

Xiangyan Li, [xiangyan_li1981@163.com;](mailto:xiangyan_li1981@163.com;) Xiaolin Tong, [tongxiaolin@vip.163.com](mailto:tongxiaolin@vip.163.com); Zeyu Wang, [zeyu781022@163.com](mailto:zeyu781022@163.com).


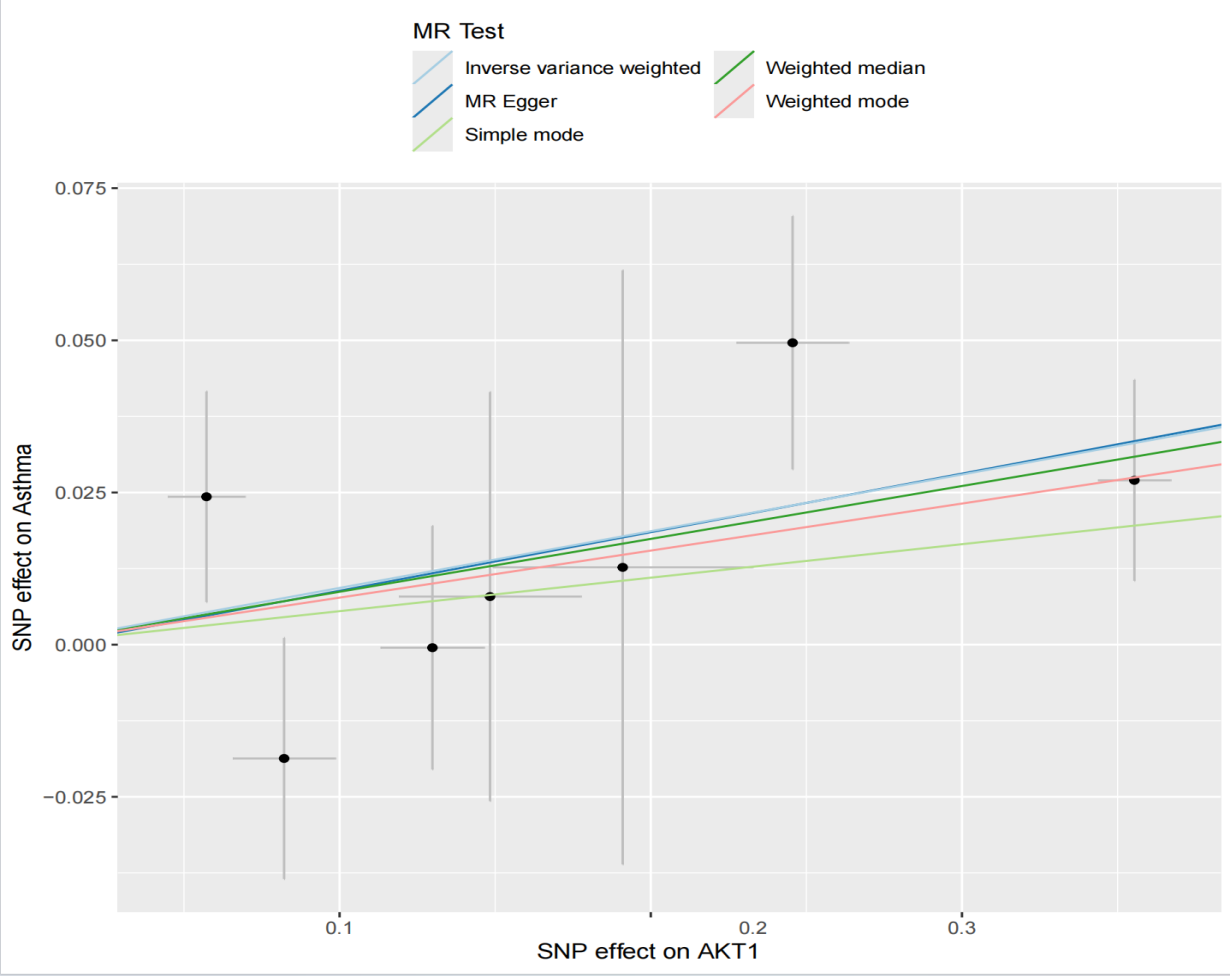


**Supplementary Figure 1:**Scatter Plots of MR Analysis for AKT1 and Asthma


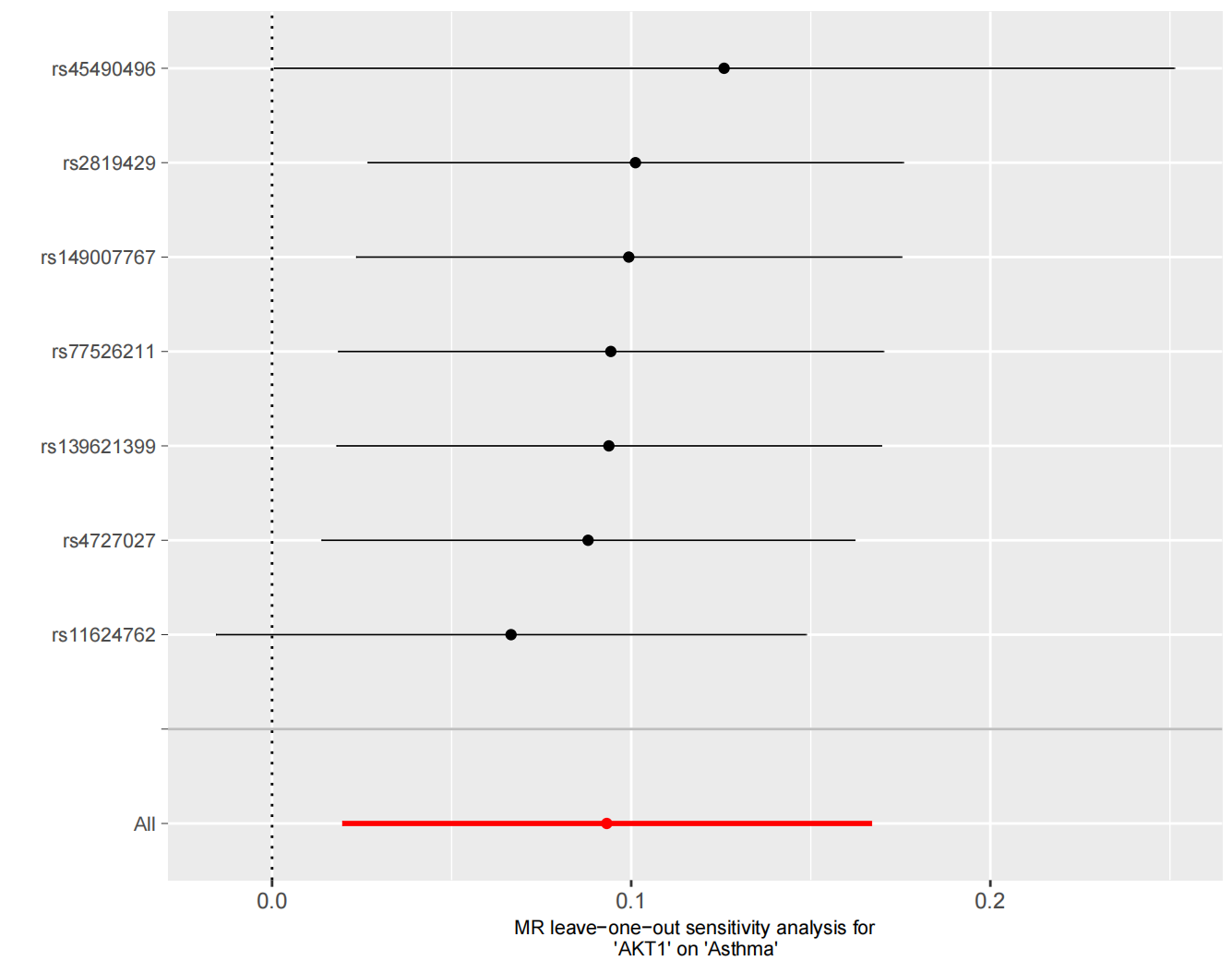


**Supplementary Figure 2:**Leave-One-Out Sensitivity Analysis for AKT1 and Asthma


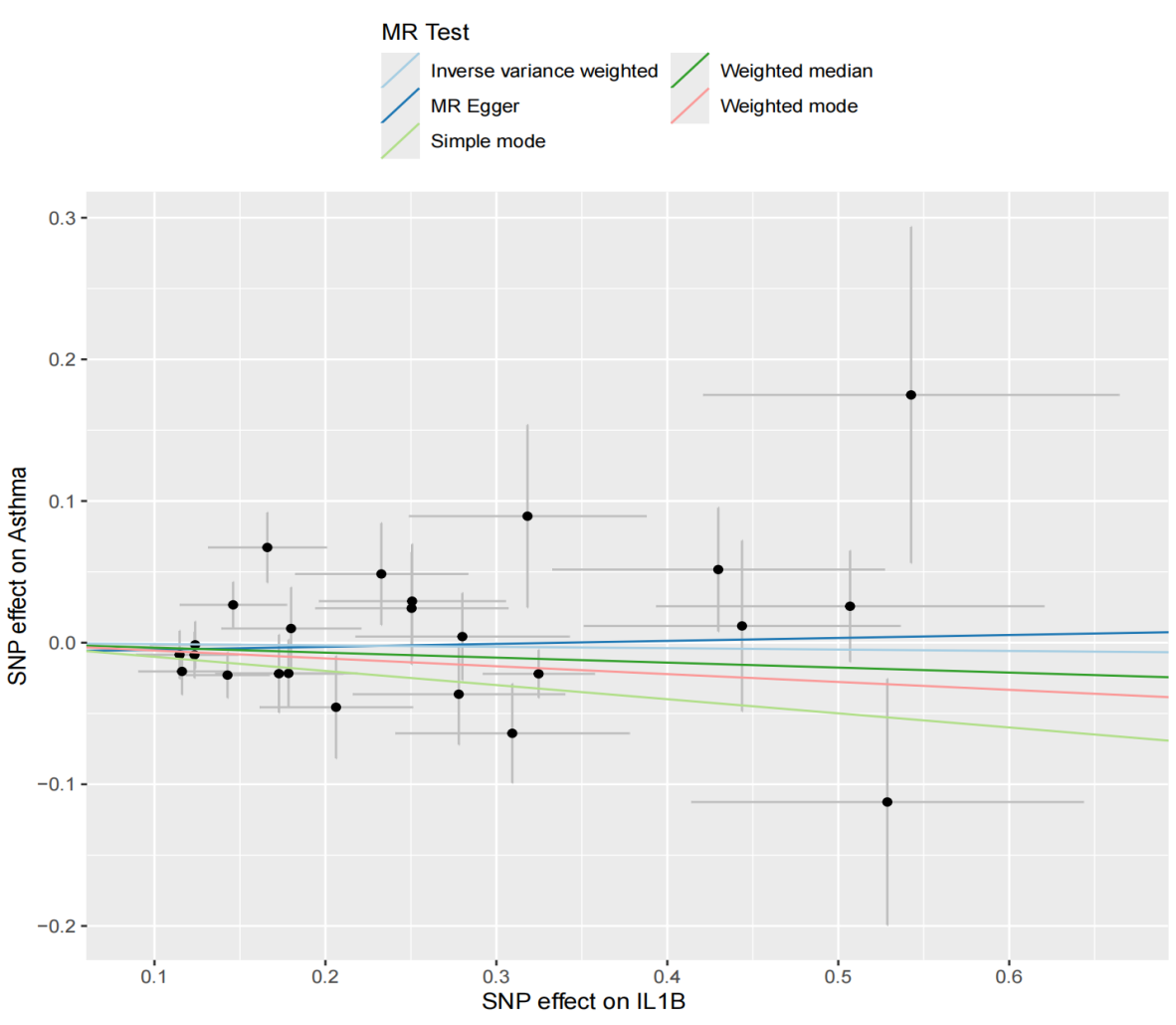


**Supplementary Figure 3:**Scatter Plots of MR Analysis for IL1B and Asthma


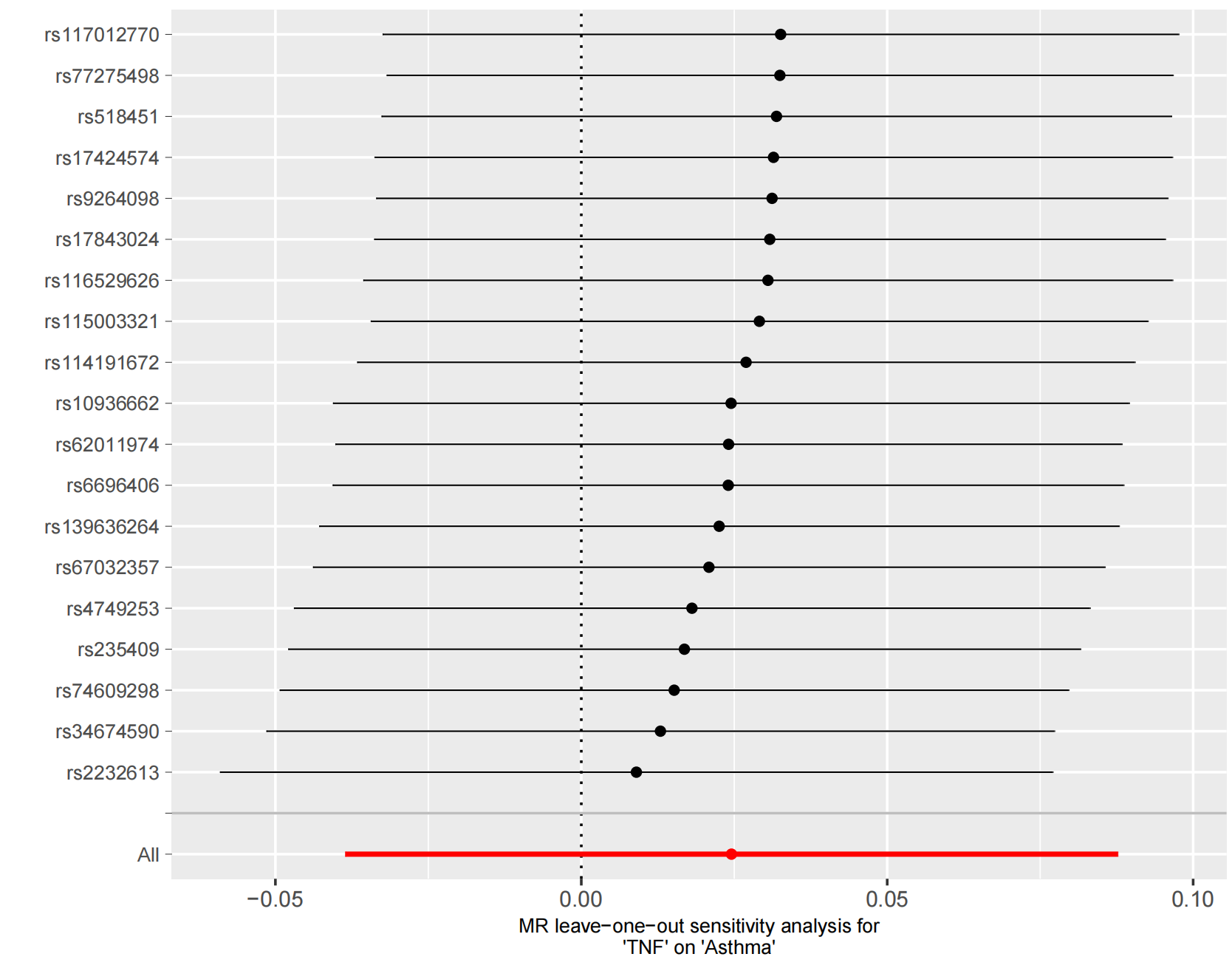


**Supplementary Figure 4:**Leave-One-Out Sensitivity Analysis for IL1B and Asthma


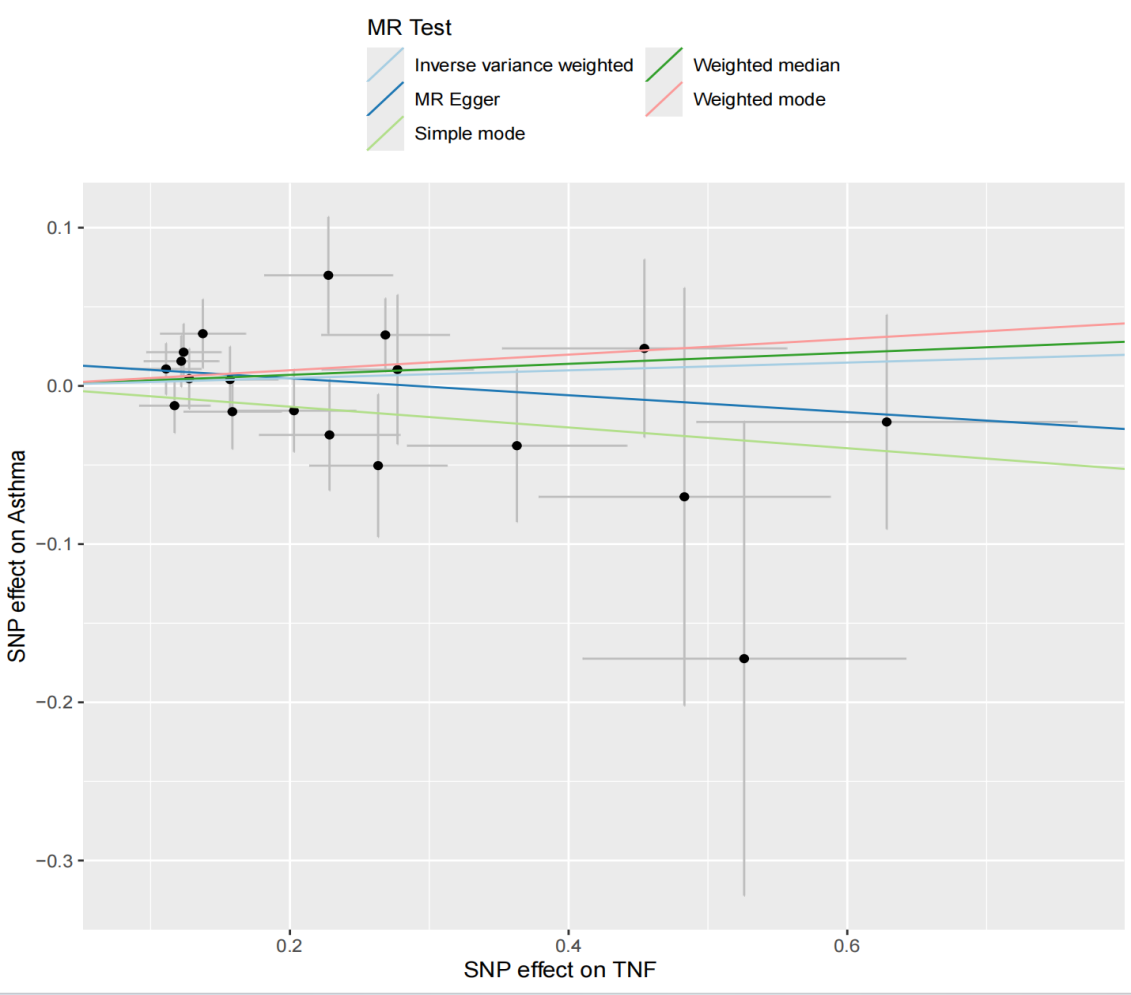


**Supplementary Figure 5:**Scatter Plots of MR Analysis for TNF and Asthma


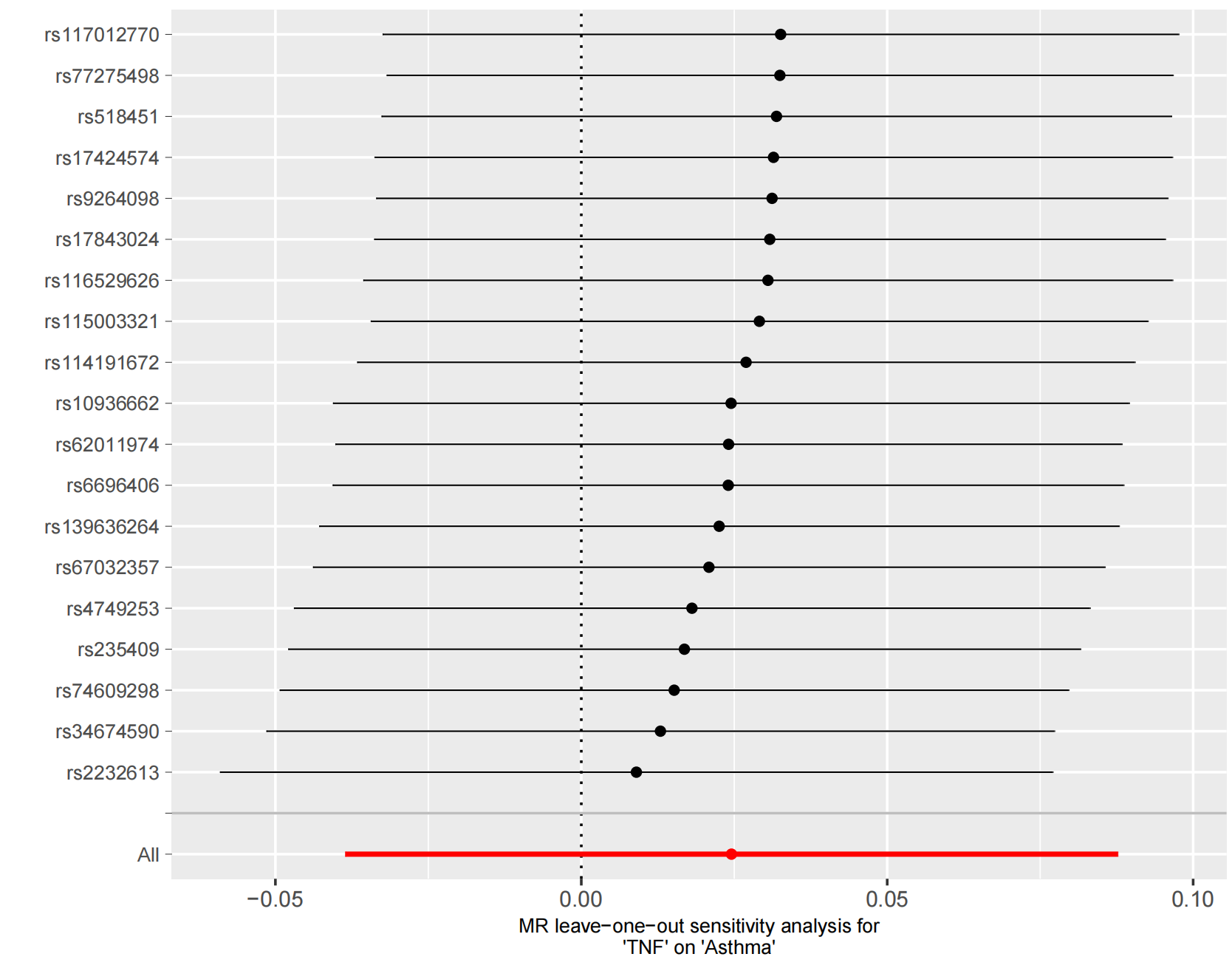


**Supplementary Figure 6:**Leave-One-Out Sensitivity Analysis for TNF and Asthma


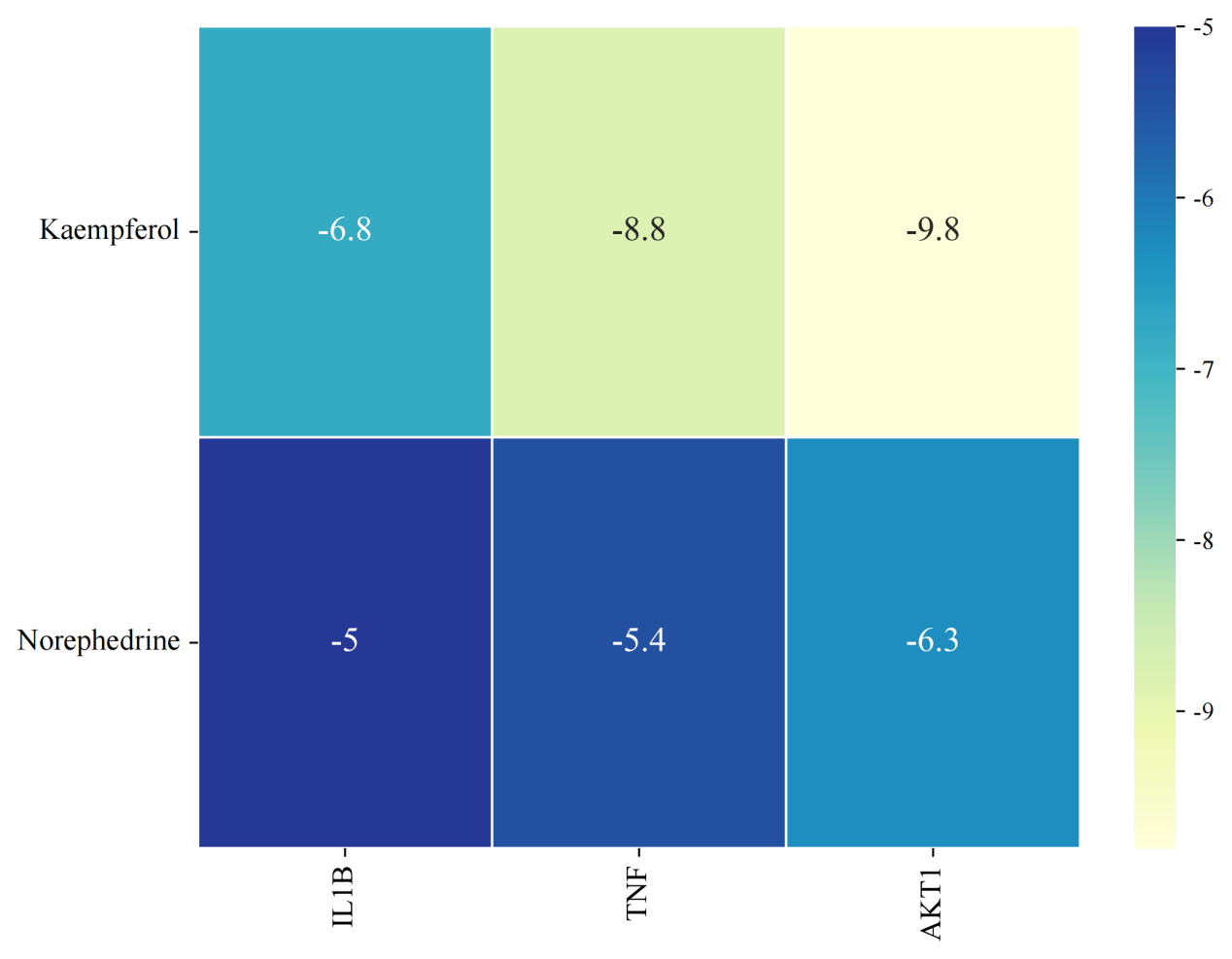


**Supplementary Figure 7:**Heat map of molecular docking
