# Supplementary material for: Yangke powder alleviates OVA-induced allergic asthma by inhibiting the PI3K/AKT/NF-κB signaling pathway
Source: Chin Med. 2025 May 26;20:69. doi: 10.1186/s13020-025-01125-x (PMC12105270; doi:10.1186/s13020-025-01125-x)
Supplement: Supplementary file 2 — Additional file 2 [file 13020_2025_1125_MOESM2_ESM.docx]

**Table S1** Primer list for real‐time PCR

| **Primer name** | **Sequence(5′–3′)** |
| --- | --- |
| NF-KB p65-mouse-F | GTATTGCTGTGCCTACCCGAAAC |
| NF-KB p65-mouse-R | GCTGAGGGATGCTGGGAAGG |
| IκB-mouse-F | GGCAATCATCCACGAAGAGAAGC |
| IκB-mouse-R | GGTTGTTCTGGAAGTTGAGGAAGG |
| IKK-mouse-F | GCAGAAGAGCGAAGTGGACATC |
| IKK-mouse-R | CAGCCGTTCAGCCAAGACAC |
| PI3K-mouse-F | GCGAAGGCAACGAGAAAGAAATTC |
| PI3K-mouse-R | TCCAACCTCCTCCTACTGTCAATG |
| AKT1-mouse-F | GGCAGGATGTGTATGAGAAGAAGC |
| AKT1-mouse-R | AGGCGGCGTGATGGTGATC |
| IL4-mouse-F | AGTTGTCATCCTGCTCTTCTTTCTC |
| IL4-mouse-R | ATGGCGTCCCTTCTCCTGTG |
| (M)TNF-α-F | TTGTCTACTCCCAGGTTCTCT |
| (M)TNF-α-R | GAGGTTGACTTTCTCCTGGTATG |
| (M)β-actin-F | CCAGCCTTCCTTCTTGGGTA |
| (M)β-actin-R | CAATGCCTGGGTACATGGTG |
| IL-10(Mouse)F | GGACAACATACTGCTAACCGACTC |
| IL-10(Mouse)R | TGGATCATTTCCGATAAGGCTTGG |
| IL-13(mouse)F | TGTTTCGCCACGGCCCCTTC |
| IL-13(mouse)R | GCAGTGCAGGCTGAGGCCAA |
| IFN-γ(mouse)F | GCTACACACTGCATCTTGGCTTTG |
| IFN-γ(mouse)R | CACTCGGATGAGCTCATTGAATGC |
| IL5-mouse-F | AGAGAAGTGTGGCGAGGAGAGAC |
| IL5-mouse-R | GCCTTCCATTGCCCACTCTGTAC |
