# Supplementary material for: Yangke powder alleviates OVA-induced allergic asthma by inhibiting the PI3K/AKT/NF-κB signaling pathway
Source: Chin Med. 2025 May 26;20:69. doi: 10.1186/s13020-025-01125-x (PMC12105270; doi:10.1186/s13020-025-01125-x)
Supplement: Supplementary file 3 — Additional file 3 [file 13020_2025_1125_MOESM3_ESM.docx]

**Table S2** The chemical compositions of Yang Kesan(YKS)

| Number | Compound name | Molecular formula | Calculated mass(MV) | m/z | *t*R/min | Reference Ion |
| --- | --- | --- | --- | --- | --- | --- |
| 1 | Nicotinic acid | C6 H5 N O2 | 123.03238 | 124.03966 | 2.13 | [M+H]+1 |
| 2 | Salicylic acid | C7 H6 O3 | 138.03061 | 137.02333 | 12.948 | [M-H]-1 |
| 3 | Methylsuccinic acid | C5 H8 O4 | 132.04121 | 131.03394 | 6.203 | [M-H]-1 |
| 4 | Nicotinamide | C6 H6 N2 O | 140.059 | 123.05572 | 2.287 | [M+H-H2O]+1 |
| 5 | Azelaic acid | C9 H16 O4 | 188.10418 | 187.09689 | 13.443 | [M-H]-1 |
| 6 | (11E,15Z)-9,10,13-trihydroxyoctadeca-11,15-dienoic acid | C18 H32 O5 | 350.20745 | 351.21472 | 15.971 | [M+H]+1 |
| 7 | Citraconic acid | C5 H6 O4 | 130.02591 | 129.01825 | 5.753 | [M-H]-1 |
| 8 | Citric acid | C6 H8 O7 | 192.02623 | 191.01895 | 2.746 | [M-H]-1 |
| 9 | Corchorifatty acid F | C18 H32 O5 | 328.22529 | 327.21802 | 15.999 | [M-H]-1 |
| 10 | Gallic acid | C7 H6 O5 | 170.0208 | 169.01352 | 5.225 | [M-H]-1 |
| 11 | D-(-)-Quinic acid | C7 H12 O6 | 210.07318 | 191.05531 | 1.447 | [M-H-H2O]-1 |
| 12 | (1S,3R,4R,5R)-1,3,4-trihydroxy-5-{[(2E)-3-(4-hydroxy-3-methoxyphenyl)prop-2-enoyl]oxy}cyclohexane-1-carboxylic acid | C17 H20 O9 | 368.11108 | 367.10379 | 11.378 | [M-H]-1 |
| 13 | 6-Hydroxycaproic acid | C6 H12 O3 | 132.07757 | 131.0703 | 10.541 | [M-H]-1 |
| 14 | Xanthurenic acid | C10 H7 N O4 | 205.03772 | 206.045 | 9.079 | [M+H]+1 |
| 15 | 9-Oxo-10(E),12(E)-octadecadienoic acid | C18 H30 O3 | 294.22005 | 295.22729 | 19.509 | [M+H]+1 |
| 16 | 3-Hydroxy-3-(methoxycarbonyl)pentanedioic acid | C7 H10 O7 | 206.04228 | 205.03497 | 5.899 | [M-H]-1 |
| 17 | D-(+)-Pipecolinic acid | C6 H11 N O2 | 129.07924 | 130.08652 | 1.496 | [M+H]+1 |
| 18 | 12-Oxo phytodienoic acid | C18 H28 O3 | 292.20415 | 293.21143 | 15.988 | [M+H]+1 |
| 19 | N-Acetyl-L-leucine | C8 H15 N O3 | 173.10567 | 174.11295 | 10.957 | [M+H]+1 |
| 20 | Suberic acid | C8 H14 O4 | 174.08851 | 173.08124 | 11.926 | [M-H]-1 |
| 21 | Isocitric acid | C6 H8 O7 | 192.02631 | 191.01906 | 1.555 | [M-H]-1 |
| 22 | 12-oxo Phytodienoic Acid | C18 H28 O3 | 292.20402 | 293.21127 | 15.122 | [M+H]+1 |
| 23 | 3-Phenyllactic acid | C9 H10 O3 | 166.06214 | 165.05493 | 11.313 | [M-H]-1 |
| 24 | 3-Hydroxy-3-(methoxycarbonyl)pentanedioic acid | C7 H10 O7 | 206.04233 | 205.03505 | 4.991 | [M-H]-1 |
| 25 | (-)-Spiculisporic acid | C17 H28 O6 | 328.18917 | 327.18195 | 21.239 | [M-H]-1 |
| 26 | Palmitic Acid | C16 H32 O2 | 273.26702 | 274.27429 | 14.922 | [M+H]+1 |
| 27 | Arachidonic acid | C20 H32 O2 | 304.24064 | 303.23337 | 22.021 | [M-H]-1 |
| 28 | 2-Furoic acid | C5 H4 O3 | 112.01444 | 111.00716 | 1.54 | [M-H]-1 |
| 29 | 3-(3,4,5-trimethoxyphenyl)propanoic acid | C12 H16 O5 | 262.08459 | 263.09186 | 11.901 | [M+H]+1 |
| 30 | 3-(3,4,5-trimethoxyphenyl)propanoic acid | C12 H16 O5 | 262.08413 | 263.0914 | 12.846 | [M+H]+1 |
| 31 | DL-Malic acid | C4 H6 O5 | 134.02037 | 133.01314 | 8.342 | [M-H]-1 |
| 32 | 3,4,5-trihydroxycyclohex-1-ene-1-carboxylic acid | C7 H10 O5 | 174.052 | 173.04472 | 11.006 | [M-H]-1 |
| 33 | L-(+)-Tartaric acid | C4 H6 O6 | 104.00984 | 149.00806 | 1.453 | [M+FA-H]-1 |
| 34 | 2-Isopropylmalic acid | C7 H12 O5 | 116.04662 | 175.06049 | 9.35 | [M-H+HAc]-1 |
| 35 | Muramic acid | C9 H17 N O7 | 219.0744 | 252.10788 | 1.559 | [M+H+MeOH]+1 |
| 36 | D-(-)-Quinic acid | C7 H12 O6 | 192.0626 | 191.05533 | 9.763 | [M-H]-1 |
| 37 | 3-[4-methyl-1-(2-methylpropanoyl)-3-oxocyclohexyl]butanoic acid | C15 H24 O4 | 290.15218 | 291.15945 | 18.085 | [M+H]+1 |
| 38 | (12Z)-9,10,11-trihydroxyoctadec-12-enoic acid | C18 H34 O5 | 352.2227 | 353.22998 | 16.51 | [M+H]+1 |
| 39 | (11E,15Z)-9,10,13-trihydroxyoctadeca-11,15-dienoic acid | C18 H32 O5 | 310.21446 | 311.22174 | 16.932 | [M+H]+1 |
| 40 | 2-(acetylamino)-3-(1H-indol-3-yl)propanoic acid | C13 H14 N2 O3 | 246.10065 | 245.09337 | 11.767 | [M-H]-1 |
| 41 | Apigenin | C15 H10 O5 | 270.05314 | 271.0603 | 15.745 | [M+H]+1 |
| 42 | Wogonin | C16 H12 O5 | 284.06888 | 285.07617 | 16.633 | [M+H]+1 |
| 43 | Baicalin | C21 H18 O11 | 446.08541 | 447.09262 | 14.475 | [M+H]+1 |
| 44 | Rutin | C27 H30 O16 | 610.15371 | 609.14642 | 12.895 | [M-H]-1 |
| 45 | Kaempferol | C15 H10 O6 | 286.0482 | 287.05548 | 12.214 | [M+H]+1 |
| 46 | Isorhamnetin | C16 H12 O7 | 316.05861 | 317.06589 | 13.672 | [M+H]+1 |
| 47 | Quercetin-3β-D-glucoside | C21 H20 O12 | 464.09594 | 463.08881 | 12.932 | [M-H]-1 |
| 48 | Cynaroside | C21 H20 O11 | 448.10103 | 449.10828 | 12.485 | [M+H]+1 |
| 49 | Tangeritin | C20 H20 O7 | 372.1209 | 373.12817 | 17.525 | [M+H]+1 |
| 50 | 5,7-dihydroxy-3-(4-hydroxyphenyl)-6-methoxy-4H-chromen-4-one | C16 H12 O6 | 300.06347 | 301.07074 | 12.372 | [M+H]+1 |
| 51 | Diosmetin | C16 H12 O6 | 300.06379 | 301.07104 | 15.813 | [M+H]+1 |
| 52 | Quercetin | C15 H10 O7 | 302.04265 | 303.04993 | 12.915 | [M+H]+1 |
| 53 | Genistein | C15 H10 O5 | 270.05327 | 269.04599 | 18.717 | [M-H]-1 |
| 54 | Nepetin | C16 H12 O7 | 316.05873 | 317.06601 | 14.037 | [M+H]+1 |
| 55 | Neohesperidin | C28 H34 O15 | 305.09491 | 609.18311 | 12.711 | [2M-H]-1 |
| 56 | Schaftoside | C26 H28 O14 | 564.14868 | 565.15594 | 11.485 | [M+H]+1 |
| 57 | Corymboside | C26 H28 O14 | 564.14887 | 565.15637 | 11.668 | [M+H]+1 |
| 58 | Luteolin | C15 H10 O6 | 286.048 | 287.05505 | 15.138 | [M+H]+1 |
| 59 | Tangeritin | C20 H20 O7 | 372.12087 | 373.12814 | 16.273 | [M+H]+1 |
| 60 | Phloretin | C15 H14 O5 | 274.08726 | 273.07999 | 27.854 | [M-H]-1 |
| 61 | 3-Methoxy-5,7,3',4'-tetrahydroxy-flavone | C16 H12 O7 | 280.0823 | 315.05173 | 14.04 | [M+Cl]-1 |
| 62 | 6-methyl-4-(morpholinomethyl)-2H-chromen-2-one | C15 H17 N O3 | 259.12139 | 260.12866 | 7.52 | [M+H]+1 |
| 63 | Eriodictyol | C15 H12 O6 | 288.06721 | 287.05994 | 19.827 | [M-H]-1 |
| 64 | Rutarin | C20 H24 O10 | 441.16438 | 442.17166 | 11.902 | [M+H]+1 |
| 65 | 5,7-dihydroxy-2-(4-hydroxy-3-methoxyphenyl)-3-{[3,4,5-trihydroxy-6-(hydroxymethyl)oxan-2-yl]oxy}-4H-chromen-4-one | C22 H22 O12 | 478.11185 | 479.1192 | 13.664 | [M+H]+1 |
| 66 | (9R,10R)-10-hydroxy-8,8-dimethyl-9-{[(2S,3R,4S,5S,6R)-3,4,5-trihydroxy-6-(hydroxymethyl)oxan-2-yl]oxy}-2H,8H,9H,10H-pyrano[2,3-h]chromen-2-one | C20 H24 O10 | 406.12663 | 407.13391 | 12.36 | [M+H]+1 |
| 67 | 5,7-dihydroxy-3-(4-hydroxyphenyl)-6-methoxy-4H-chromen-4-one | C16 H12 O6 | 300.06366 | 301.07086 | 14.99 | [M+H]+1 |
| 68 | 6-hydroxy-3-(3-hydroxy-4-{[3,4,5-trihydroxy-6-({[3,4,5-trihydroxy-6-(hydroxymethyl)oxan-2-yl]oxy}methyl)oxan-2-yl]oxy}phenyl)-5,7-dimethoxy-4H-chromen-4-one | C29 H34 O17 | 654.18052 | 655.18762 | 12.114 | [M+H]+1 |
| 69 | 5-hydroxy-2-(4-hydroxy-3-methoxyphenyl)-6-methoxy-7-{[(2S,3R,4S,5S,6R)-3,4,5-trihydroxy-6-(hydroxymethyl)oxan-2-yl]oxy}-4H-chromen-4-one | C23 H24 O12 | 492.12758 | 493.13486 | 13.657 | [M+H]+1 |
| 70 | (2S,3S,4S,5R,6S)-3,4,5-trihydroxy-6-[(5-hydroxy-8-methoxy-4-oxo-2-phenyl-4H-chromen-7-yl)oxy]oxane-2-carboxylic acid | C22 H20 O11 | 460.10075 | 461.10791 | 15.258 | [M+H]+1 |
| 71 | 5-hydroxy-2-(3-hydroxy-4-methoxyphenyl)-7-{[(2S,3R,4S,5S,6R)-3,4,5-trihydroxy-6-({[(2R,3R,4R,5R,6S)-3,4,5-trihydroxy-6-methyloxan-2-yl]oxy}methyl)oxan-2-yl]oxy}-4H-chromen-4-one | C28 H32 O15 | 608.17504 | 609.18231 | 13.364 | [M+H]+1 |
| 72 | (2Z)-6-hydroxy-2-[(4-hydroxy-3-methoxyphenyl)methylidene]-2,3-dihydro-1-benzofuran-3-one | C16 H12 O5 | 284.06892 | 285.07617 | 14.423 | [M+H]+1 |
| 73 | (4S)-4-hydroxy-3,5,5-trimethyl-4-[(1E)-3-{[(2R,3R,4S,5S,6R)-3,4,5-trihydroxy-6-(hydroxymethyl)oxan-2-yl]oxy}but-1-en-1-yl]cyclohex-2-en-1-one | C19 H30 O8 | 386.19452 | 431.19266 | 10.492 | [M+FA-H]-1 |
| 74 | (1S)-1,5-Anhydro-2-O-(6-deoxy-α-L-mannopyranosyl)-1-[5,7-dihydroxy-2-(4-hydroxyphenyl)-4-oxo-4H-chromen-6-yl]-D-glucitol | C27 H30 O14 | 546.13794 | 579.17145 | 12.519 | [M+H+MeOH]+1 |
| 75 | (2S,3S,4R,5R)-4-hydroxy-2,5-bis(hydroxymethyl)-2-{[(2R,3R,4S,5S,6R)-3,4,5-trihydroxy-6-(hydroxymethyl)oxan-2-yl]oxy}oxolan-3-yl (2E)-3-(4-hydroxy-3,5-dimethoxyphenyl)prop-2-enoate | C23 H32 O15 | 530.16442 | 531.17169 | 12.706 | [M+H]+1 |
| 76 | (1S,4aS,7aS)-7-({[(2E)-3-phenylprop-2-enoyl]oxy}methyl)-1-{[(2S,3R,4S,5S,6R)-3,4,5-trihydroxy-6-(hydroxymethyl)oxan-2-yl]oxy}-1H,4aH,5H,7aH-cyclopenta[c]pyran-4-carboxylic acid | C25 H28 O11 | 542.12535 | 543.13263 | 1.591 | [M+H]+1 |
| 77 | 5,7-dihydroxy-2-(3-hydroxy-4-methoxyphenyl)-3,6-dimethoxy-4H-chromen-4-one | C18 H16 O8 | 360.08407 | 361.09125 | 12.852 | [M+H]+1 |
| 78 | (9R,10R)-10-(acetyloxy)-8,8-dimethyl-2-oxo-2H,8H,9H,10H-pyrano[2,3-h]chromen-9-yl 3-methylbut-2-enoate | C21 H22 O7 | 408.11778 | 409.12506 | 23.062 | [M+H]+1 |
| 79 | 5,7-Dihydroxy-2-(4-hydroxyphenyl)-4-oxo-4H-chromen-3-yl 6-O-(6-deoxyhexopyranosyl)hexopyranoside | C27 H30 O15 | 594.15943 | 593.15198 | 13.604 | [M-H]-1 |
| 80 | Pseudoephedrine | C10 H15 N O | 165.11546 | 148.11214 | 6.002 | [M+H-H2O]+1 |
| 81 | Hypoxanthine | C5 H4 N4 O | 136.0387 | 137.04597 | 5.347 | [M+H]+1 |
| 82 | Sinapine | C16 H23 N O5 | 309.15709 | 310.16437 | 8.331 | [M+H]+1 |
| 83 | Hordenine | C10 H15 N O | 133.0894 | 166.12289 | 3.35 | [M+H+MeOH]+1 |
| 84 | 6-Methylquinoline | C10 H9 N | 143.07373 | 144.08101 | 8.632 | [M+H]+1 |
| 85 | Trigonelline | C7 H7 N O2 | 137.04781 | 138.05508 | 1.435 | [M+H]+1 |
| 86 | 7-hydroxy-6-methoxy-2H-chromen-2-one | C10 H8 O4 | 192.0425 | 193.04991 | 11.507 | [M+H]+1 |
| 87 | Betaine | C5 H11 N O2 | 117.07926 | 118.08656 | 1.42 | [M+H]+1 |
| 88 | Choline | C5 H13 N O | 103.10027 | 104.10754 | 1.585 | [M+H]+1 |
| 89 | DL-Stachydrine | C7 H13 N O2 | 111.06858 | 144.10207 | 1.57 | [M+H+MeOH]+1 |
| 90 | Berberine | C20 H17 N O4 | 303.08962 | 336.12311 | 11.37 | [M+H+MeOH]+1 |
| 91 | trans-3-Indoleacrylic acid | C11 H9 N O2 | 187.06376 | 188.07103 | 7.35 | [M+H]+1 |
| 92 | Norephedrine | C9 H13 N O | 151.09984 | 152.10712 | 4.66 | [M+H]+1 |
| 93 | 2-Oxindole | C8 H7 N O | 151.06352 | 134.06024 | 9.965 | [M+H-H2O]+1 |
| 94 | Sinomenine | C19 H23 N O4 | 329.15907 | 330.16635 | 5.118 | [M+H]+1 |
| 95 | [(3R,5R,6S,8S)-3-(β-D-Glucopyranosyloxy)-6-hydroxy-8-methyl-9,10-dioxatetracyclo[4.3.1.02,5 03,8]dec-2-yl]methyl benzoate | C23 H28 O11 | 480.16358 | 481.17093 | 10.063 | [M+H]+1 |
| 96 | 7,7-dimethyl-3-spiro(4,4,-dimethyl-2,6-dioxocyclohexyl)-1,2,3,4,5,6,7,8-octahydro-5-quinolinone | C18 H25 N O3 | 303.18385 | 304.19113 | 14.464 | [M+H]+1 |
| 97 | 6-[(4-methylphenyl)sulfonyl]-6-azabicyclo[3.2.1]octane | C14 H19 N O2 S | 303.07189 | 304.07916 | 1.303 | [M+H]+1 |
| 98 | N-(4-heptylbenzoyl)-N'-(6-methylpyridin-2-yl)thiourea | C21 H27 N3 O S | 369.19432 | 370.2016 | 17.876 | [M+H]+1 |
| 99 | Chlorogenic acid | C16 H18 O9 | 354.0952 | 353.0878 | 9.768 | [M-H]-1 |
| 100 | Shogaol | C17 H24 O3 | 276.17273 | 277.17999 | 17.573 | [M+H]+1 |
| 101 | Schisandrin | C24 H32 O7 | 432.21436 | 415.211 | 16.183 | [M+H-H2O]+1 |
| 102 | 4,5-Dicaffeoylquinic acid | C25 H24 O12 | 258.06354 | 515.11981 | 13.353 | [2M-H]-1 |
| 103 | Neochlorogenic acid | C16 H18 O9 | 354.09526 | 353.08798 | 8.197 | [M-H]-1 |
| 104 | Sinapinic acid | C11 H12 O5 | 174.03215 | 207.06563 | 11.956 | [M+H+MeOH]+1 |
| 105 | Gentisic acid | C7 H6 O4 | 108.02026 | 153.01846 | 7.35 | [M+FA-H]-1 |
| 106 | 6-Gingerol | C17 H26 O4 | 276.17254 | 277.17981 | 16.17 | [M+H]+1 |
| 107 | Trifolin | C21 H20 O11 | 448.10136 | 447.09393 | 13.599 | [M-H]-1 |
| 108 | Vanillin | C8 H8 O3 | 152.04779 | 153.05507 | 10.625 | [M+H]+1 |
| 109 | Apocynin | C9 H10 O3 | 166.0633 | 167.07057 | 7.677 | [M+H]+1 |
| 110 | 3-(3,4-dihydroxyphenyl)propanoic acid | C9 H10 O4 | 164.04767 | 182.08151 | 2.995 | [M+NH4]+1 |
| 111 | Pyrogallol | C6 H6 O3 | 126.03201 | 127.03928 | 1.702 | [M+H]+1 |
| 112 | Catechin | C15 H14 O6 | 308.09341 | 289.07556 | 17.938 | [M-H-H2O]-1 |
| 113 | 4-Methoxybenzaldehyde | C8 H8 O2 | 136.05245 | 137.05972 | 16.17 | [M+H]+1 |
| 114 | Piceatannol | C14 H12 O4 | 262.08442 | 243.06656 | 12.127 | [M-H-H2O]-1 |
| 115 | Syringic acid | C9 H10 O5 | 198.05235 | 197.04507 | 11.202 | [M-H]-1 |
| 116 | Caffeic acid | C9 H8 O4 | 180.04172 | 179.03444 | 12.227 | [M-H]-1 |
| 117 | (1S,3R,4S,5R)-3,5-bis({[(2E)-3-(3,4-dihydroxyphenyl)prop-2-enoyl]oxy})-1,4-dihydroxycyclohexane-1-carboxylic acid | C25 H24 O12 | 516.12772 | 517.13501 | 13.337 | [M+H]+1 |
| 118 | (1r,3R,4s,5S)-4-{[(2E)-3-(3,4-dihydroxyphenyl)prop-2-enoyl]oxy}-1,3,5-trihydroxycyclohexane-1-carboxylic acid | C16 H18 O9 | 354.09547 | 353.08823 | 12.392 | [M-H]-1 |
| 119 | α-Cyano-3-hydroxycinnamic acid | C10 H7 N O3 | 189.04211 | 188.03484 | 10.704 | [M-H]-1 |
| 120 | 5-[4-(3-hydroxy-4-methoxyphenyl)-hexahydrofuro[3,4-c]furan-1-yl]-2-methoxyphenol | C20 H22 O6 | 340.13179 | 341.13898 | 13.64 | [M+H]+1 |
| 121 | Asiatic acid | C30 H48 O5 | 488.3509 | 487.34351 | 18.233 | [M-H]-1 |
| 122 | (-)-Caryophyllene oxide | C15 H24 O | 220.18295 | 221.19023 | 15.824 | [M+H]+1 |
| 123 | Citral | C10 H16 O | 152.12032 | 153.12758 | 13.222 | [M+H]+1 |
| 124 | (±)-Abscisic acid | C15 H20 O4 | 286.11685 | 287.12411 | 5.285 | [M+H]+1 |
| 125 | Eucalyptol | C10 H18 O | 154.13592 | 137.13263 | 12.754 | [M+H-H2O]+1 |
| 126 | Sedanolide | C12 H18 O2 | 194.13082 | 227.16423 | 16.42 | [M+H+MeOH]+1 |
| 127 | 12,12-dimethyl-13-[(3-methylbut-2-enoyl)oxy]-4-oxo-3,11-dioxatricyclo[8.4.0.0Â?,â·]tetradeca-1,5,7,9-tetraen-14-yl 3-methylbut-2-enoate | C24 H26 O7 | 443.1949 | 444.20218 | 18.597 | [M+H]+1 |
| 128 | 6-O-Hexopyranosyl-1-O-[19-hydroxy-28-oxo-3-(pentopyranosyloxy)olean-12-en-28-yl]hexopyranose | C47 H76 O18 | 974.50978 | 973.5025 | 16.909 | [M-H]-1 |
| 129 | 6-formyl-10-(hydroxymethyl)-5-methoxy-3-methylidene-2-oxo-2H,3H,3aH,4H,5H,8H,9H,11aH-cyclodeca[b]furan-4-yl 2-methylbutanoate | C21 H28 O7 | 414.16814 | 415.17542 | 17.051 | [M+H]+1 |
| 130 | (1aR,1bR,2R,3R,7R,7aS)-1b,2-dimethyl-7a-(prop-1-en-2-yl)-1aH,1bH,2H,3H,4H,5H,7H,7aH-naphtho[1,2-b]oxirene-3,7-diol | C15 H22 O3 | 232.14661 | 233.15384 | 18.497 | [M+H]+1 |
| 131 | Adenosine | C10 H13 N5 O4 | 267.09677 | 268.10403 | 4.587 | [M+H]+1 |
| 132 | D-Glucosamine | C6 H13 N O5 | 179.07939 | 218.04254 | 1.146 | [M+K]+1 |
| 133 | D-(+)-Maltose | C12 H22 O11 | 364.09795 | 365.10522 | 1.365 | [M+H]+1 |
| 134 | albiflorin | C23 H28 O11 | 466.14818 | 525.16205 | 10.075 | [M-H+HAc]-1 |
| 135 | L-Iditol | C6 H14 O6 | 182.07935 | 183.08662 | 1.659 | [M+H]+1 |
| 136 | 2'-O-Methyladenosine | C11 H15 N5 O4 | 281.11272 | 282.12 | 5.978 | [M+H]+1 |
| 137 | Sibiricose A1 | C23 H32 O15 | 548.17537 | 547.16803 | 10.78 | [M-H]-1 |
| 138 | 4-Acetyl-3-hydroxy-5-methylphenyl β-D-glucopyranoside | C15 H20 O8 | 328.11632 | 373.11447 | 9.132 | [M+FA-H]-1 |
| 139 | Uridine | C9 H12 N2 O6 | 244.06976 | 243.06238 | 3.653 | [M-H]-1 |
| 140 | Kanosamine | C6 H13 N O5 | 161.06887 | 162.07614 | 1.177 | [M+H]+1 |
| 141 | α,α-Trehalose | C12 H22 O11 | 342.11612 | 377.08551 | 1.405 | [M+Cl]-1 |
| 142 | Guanosine monophosphate (GMP) | C10 H14 N5 O8 P | 363.05855 | 362.05127 | 6.84 | [M-H]-1 |
| 143 | Thymidine | C10 H14 N2 O5 | 242.09061 | 241.0831 | 6.557 | [M-H]-1 |
| 144 | (6,6-Dimethylbicyclo[3.1.1]hept-2-yl)methyl 6-O-[(2R,3R,4R)-3,4-dihydroxy-4-(hydroxymethyl)tetrahydro-2-furanyl]-β-D-glucopyranoside | C21 H36 O10 | 465.25795 | 466.26523 | 15.055 | [M+H]+1 |
| 145 | 2-(4-Methyl-3-cyclohexen-1-yl)-2-propanyl 6-O-(6-deoxy-α-L-mannopyranosyl)-β-D-glucopyranoside | C22 H38 O10 | 508.25255 | 507.24527 | 15.335 | [M-H]-1 |
| 146 | (2R)-2-(β-D-Glucopyranosyloxy)-2-phenylacetamide | C14 H19 N O7 | 335.10091 | 336.10818 | 8.511 | [M+H]+1 |
| 147 | 2-(2-Oxo-8,9-dihydro-2H-furo[2,3-h]chromen-8-yl)-2-propanyl beta-D-glucopyranoside | C20 H24 O9 | 454.14812 | 453.14084 | 12.583 | [M-H]-1 |
| 148 | Adenine | C5 H5 N5 | 118.02816 | 136.06198 | 4.586 | [M+NH4]+1 |
| 149 | Cuminaldehyde | C10 H12 O | 148.08901 | 149.09628 | 17.176 | [M+H]+1 |
| 150 | tert-Butyl N-[1-(aminocarbonyl)-3-methylbutyl]carbamate | C11 H22 N2 O3 | 230.1633 | 231.17059 | 6.279 | [M+H]+1 |
| 151 | Palmitoyl ethanolamide | C18 H37 N O2 | 299.28297 | 300.29025 | 21.43 | [M+H]+1 |
| 152 | Lipoxin B4 | C20 H32 O5 | 352.22529 | 351.21802 | 16.581 | [M-H]-1 |
| 153 | 2-Amino-1,3,4-octadecanetriol | C18 H39 N O3 | 317.2932 | 318.30048 | 14.977 | [M+H]+1 |
| 154 | Ethyl palmitoleate | C18 H34 O2 | 282.25641 | 283.26367 | 21.538 | [M+H]+1 |
| 155 | 4-Methoxybenzaldehyde | C8 H8 O2 | 136.05255 | 137.05983 | 17.576 | [M+H]+1 |
| 156 | 2-(4-chlorophenoxy)-N-(2-oxo-3-azepanyl)nicotinamide | C18 H18 Cl N3 O3 | 359.10127 | 360.10855 | 11.114 | [M+H]+1 |
| 157 | 12-oxo Phytodienoic Acid | C18 H28 O3 | 292.20433 | 293.21161 | 21.034 | [M+H]+1 |
| 158 | D-Panthenol | C9 H19 N O4 | 227.11624 | 228.12352 | 11.952 | [M+H]+1 |
| 159 | 5-Methoxyindoleacetic acid | C11 H11 N O3 | 205.07407 | 206.08144 | 8.329 | [M+H]+1 |
| 160 | Guanine | C5 H5 N5 O | 134.02314 | 152.05696 | 5.328 | [M+NH4]+1 |
| 161 | 1-O-[(2alpha,3beta,5xi,9xi,18xi)-2,3,19-Trihydroxy-28-oxours-12-en-28-yl]-beta-D-glucopyranose | C36 H58 O10 | 696.40974 | 695.40247 | 15.898 | [M-H]-1 |
| 162 | 4-(3,4-dihydro-2H-1,5-benzodioxepin-7-ylmethyl)-1lambda~6~,4-thiazinane-1,1-dione | C14 H19 N O4 S | 319.08544 | 320.09271 | 10.733 | [M+H]+1 |
| 163 | 4-(4-methoxyphenyl)-6-pyridin-4-yl-1,3,5-triazin-2(3H)-one | C15 H12 N4 O2 | 318.05184 | 319.05911 | 27.779 | [M+H]+1 |
| 164 | Methoxsalen | C12 H8 O4 | 216.04254 | 217.04982 | 14.06 | [M+H]+1 |
| 165 | 7-Hydroxycoumarine | C9 H6 O3 | 162.03192 | 163.0392 | 11.35 | [M+H]+1 |
| 166 | Columbianetin | C14 H14 O4 | 246.08901 | 534.21173 | 13.001 | [2M+ACN+H]+1 |
| 167 | Psoralen | C11 H6 O3 | 186.03208 | 187.03935 | 13.85 | [M+H]+1 |
| 168 | 4-Hydroxycoumarin | C9 H6 O3 | 162.0309 | 161.02362 | 11.343 | [M-H]-1 |
| 169 | Fraxetin | C10 H8 O5 | 208.03751 | 209.04518 | 10.761 | [M+H]+1 |
| 170 | Murrangatin | C15 H16 O5 | 276.10362 | 275.09634 | 20.119 | [M-H]-1 |
| 171 | Coumarin | C9 H6 O2 | 146.0371 | 147.04437 | 10.983 | [M+H]+1 |
| 172 | 5-hydroxy-2-(4-hydroxyphenyl)-6-methoxy-7-{[(2S,3R,4S,5S,6R)-3,4,5-trihydroxy-6-(hydroxymethyl)oxan-2-yl]oxy}-4H-chromen-4-one | C22 H22 O11 | 462.11711 | 463.12442 | 13.558 | [M+H]+1 |
| 173 | (2S)-2-(2-hydroxypropan-2-yl)-2H,3H,7H-furo[3,2-g]chromen-7-one | C14 H14 O4 | 246.08943 | 247.09676 | 12.635 | [M+H]+1 |
| 174 | 5-hydroxy-3-(5-hydroxy-2,4-dimethoxyphenyl)-6-methoxy-7-{[3,4,5-trihydroxy-6-(hydroxymethyl)oxan-2-yl]oxy}-4H-chromen-4-one | C24 H26 O13 | 522.13742 | 523.14453 | 12.851 | [M+H]+1 |
