# Supplementary material for: Yangke powder alleviates OVA-induced allergic asthma by inhibiting the PI3K/AKT/NF-κB signaling pathway
Source: Chin Med. 2025 May 26;20:69. doi: 10.1186/s13020-025-01125-x (PMC12105270; doi:10.1186/s13020-025-01125-x)
Supplement: Supplementary file 4 — Additional file 4 [file 13020_2025_1125_MOESM4_ESM.docx]

**Table S3** The Blood-Absorbed components of Yang Kesan(YKS)

| Number | Compound name | Molecular formula | Calc. MW | m/z | *t*R/min | Reference Ion |
| --- | --- | --- | --- | --- | --- | --- |
| 1 | Nicotinic acid | C6 H5 N O2 | 123.03238 | 124.03896 | 2.03 | [M+H]+1 |
| 2 | Adenosine | C10 H13 N5 O4 | 267.09677 | 268.10433 | 4.117 | [M+H]+1 |
| 3 | Pseudoephedrine | C10 H15 N O | 165.1154 | 148.11212 | 5.686 | [M+H-H2O]+1 |
| 4 | Hypoxanthine | C5 H4 N4 O | 136.03868 | 137.04596 | 2.877 | [M+H]+1 |
| 5 | Apigenin | C15 H10 O5 | 270.05314 | 271.2673 | 15.651 | [M+H]+1 |
| 6 | Chlorogenic acid | C16 H18 O9 | 354.0952 | 353.0921 | 9.178 | [M-H]-1 |
| 7 | Wogonin | C16 H12 O5 | 284.06888 | 285.07623 | 16.579 | [M+H]+1 |
| 8 | Baicalin | C21 H18 O11 | 446.08541 | 447.09260 | 14.476 | [M+H]+1 |
| 9 | Adenine | C5 H5 N5 | 135.05469 | 136.06197 | 1.538 | [M+H]+1 |
| 10 | Methoxsalen | C12 H8 O4 | 216.04247 | 217.04974 | 14.971 | [M+H]+1 |
| 11 | Rutin | C27 H30 O16 | 610.15426 | 609.14691 | 12.217 | [M-H]-1 |
| 12 | Salicylic acid | C7 H6 O3 | 138.03061 | 137.02352 | 12.936 | [M-H]-1 |
| 13 | Sinapine | C16 H23 N O5 | 309.15709 | 310.16537 | 8.031 | [M+H]+1 |
| 14 | Kaempferol | C15 H10 O6 | 286.04839 | 287.05566 | 13.594 | [M+H]+1 |
| 15 | Isorhamnetin | C16 H12 O7 | 316.05861 | 317.23224 | 13.591 | [M+H]+1 |
| 16 | 6-Methylquinoline | C10 H9 N | 143.07373 | 144.08121 | 8.467 | [M+H]+1 |
| 17 | Nicotinamide | C6 H6 N2 O | 140.059 | 123.05673 | 2.185 | [M+H-H2O]+1 |
| 18 | Azelaic acid | C9 H16 O4 | 188.10418 | 187.28735 | 13.805 | [M-H]-1 |
| 19 | Shogaol | C17 H24 O3 | 276.17273 | 277.18677 | 17.692 | [M+H]+1 |
| 20 | Trigonelline | C7 H7 N O2 | 137.04781 | 138.221 | 1.42 | [M+H]+1 |
| 21 | Cynaroside | C21 H20 O11 | 448.10103 | 449.10896 | 12.481 | [M+H]+1 |
| 22 | Citric acid | C6 H8 O7 | 192.02623 | 191.01398 | 2.734 | [M-H]-1 |
| 23 | Diosmetin | C16 H12 O6 | 300.06379 | 301.07597 | 15.729 | [M+H]+1 |
| 24 | Quercetin | C15 H10 O7 | 302.04265 | 303.04582 | 12.076 | [M+H]+1 |
| 25 | Schisandrin | C24 H32 O7 | 432.21436 | 415.2139 | 16.044 | [M+H-H2O]+1 |
| 26 | Gallic acid | C7 H6 O5 | 170.0208 | 169.01259 | 5.012 | [M-H]-1 |
| 27 | Betaine | C5 H11 N O2 | 117.07926 | 118.08661 | 1.555 | [M+H]+1 |
| 28 | Choline | C5 H13 N O | 103.10013 | 104.10741 | 1.314 | [M+H]+1 |
| 29 | 4,5-Dicaffeoylquinic acid | C25 H24 O12 | 516.12692 | 515.11957 | 12.404 | [M-H]-1 |
| 30 | albiflorin | C23 H28 O11 | 526.16951 | 525.16223 | 10.828 | [M-H]-1 |
| 31 | Xanthurenic acid | C10 H7 N O4 | 205.03772 | 206.042 | 9.829 | [M+H]+1 |
| 32 | Psoralen | C11 H6 O3 | 186.0322 | 187.03947 | 11.7 | [M+H]+1 |
| 33 | Berberine | C20 H17 N O4 | 335.11662 | 336.1239 | 10.87 | [M+H]+1 |
| 34 | Genistein | C15 H10 O5 | 270.05327 | 269.14573 | 17.907 | [M-H]-1 |
| 35 | Neochlorogenic acid | C16 H18 O9 | 354.09526 | 353.87098 | 8.076 | [M-H]-1 |
| 36 | Norephedrine | C9 H13 N O | 151.09984 | 152.07731 | 4.02 | [M+H]+1 |
| 37 | Luteolin | C15 H10 O6 | 286.048 | 287.05515 | 15.130 | [M+H]+1 |
| 38 | Vanillin | C8 H8 O3 | 152.04779 | 153.27553 | 10.703 | [M+H]+1 |
| 39 | 3-Phenyllactic acid | C9 H10 O3 | 166.06214 | 165.05498 | 11.541 | [M-H]-1 |
| 40 | Palmitic Acid | C16 H32 O2 | 273.26702 | 274.27432 | 14.927 | [M+H]+1 |
| 41 | Arachidonic acid | C20 H32 O2 | 304.24064 | 303.23323 | 22.011 | [M-H]-1 |
| 42 | Pyrogallol | C6 H6 O3 | 126.03201 | 127.03924 | 1.708 | [M+H]+1 |
| 43 | Eriodictyol | C15 H12 O6 | 288.06721 | 287.05997 | 19.821 | [M-H]-1 |
| 44 | Fraxetin | C10 H8 O5 | 208.03703 | 207.02972 | 11.99 | [M-H]-1 |
| 45 | Sinomenine | C19 H23 N O4 | 329.15907 | 330.16536 | 5.108 | [M+H]+1 |
| 46 | Eucalyptol | C10 H18 O | 136.12544 | 137.13272 | 15.416 | [M+H]+1 |
| 47 | Syringic acid | C9 H10 O5 | 198.05235 | 197.04313 | 11.192 | [M-H]-1 |
| 48 | Coumarin | C9 H6 O2 | 146.03704 | 147.04431 | 9.22 | [M+H]+1 |
| 49 | Caffeic acid | C9 H8 O4 | 180.0448 | 179.03795 | 11.299 | [M-H]-1 |
